# Enhanced operation of PVWPS based on advanced soft computing optimization techniques

**DOI:** 10.1038/s41598-024-80894-1

**Published:** 2024-11-27

**Authors:** Mahmoud M. Elymany, Mohamed A. Enany, Hamid Metwally, Ahmed A. Shaier

**Affiliations:** Electrical Power and Machines Department, Faculty of Engineering, Zagazig University, Zagazig, 44519 Egypt

**Keywords:** Soft computing, Gorilla troop algorithm (GTO), Honey badger algorithm (HBA), Snake algorithm (SAO), ANFIS, MATLAB simulation, Electrical and electronic engineering, Solar energy, Photovoltaics

## Abstract

This study introduces three soft computing (SC) optimization algorithms aimed at enhancing the efficiency of photovoltaic water pumping systems (PVWPS). These algorithms include the Gorilla Troop Algorithm (GTO), Honey Badger Algorithm (HBA), and Snake Algorithm (SAO). The goal of the SC optimizers is to maximize the output power of the PV array (*P*_*PV*_) and enhance the efficiency of the DC motor (*η*), thereby optimizing the water flow rate (*Q*) of the pumping system. The analytical modeling approach proposed in this study involves forecasting the optimal duty cycle (*D*_*op*_) for a buck-boost converter, taking into account variables such as solar radiation (*G*) and ambient temperature (*T*). A comparative analysis is conducted between the suggested SC optimizers and analytical modeling. MATLAB simulation is employed to explore an adaptive neuro-fuzzy inference system (ANFIS) trained for the proposed system. The objective is to assess system performance and accuracy. Findings indicate a strong convergence between the analytical model and the simulation model utilizing SC optimizers. Moreover, the neuro-fuzzy system trained offline, coupled with the proposed SC optimizers, demonstrates superior performance compared to traditional control methods like perturb and observe (P&O) and incremental conductance (IC). This superiority is evident across various metrics including motor efficiency (*η*), photovoltaic (PV) output power (*P*_*PV*_), water flow rate (*Q*), and time response.

**Supplementary Information:**

The online version contains supplementary material available at 10.1038/s41598-024-80894-1.

## Introduction

PV systems are recognized as crucial solutions for delivering clean and sustainable energy^1–3^. Integrating PV systems with other components to create photovoltaic water pumping systems (PVWPS) offers a solution for providing water in desert and remote regions. PVWPS present numerous advantages, including emission-free operation, cost-effectiveness, extended lifespan, and enhanced water flow. Moreover, they incur low maintenance expenses and operate silently during generation, making them a practical and efficient choice for water pumping in remote areas^4,5^. Nonetheless, PVWPS face significant drawbacks, including higher initial capital costs in comparison to traditional fuel-based water pumping systems, the need for regular maintenance, and limited pumping capacity. Consequently, they may not be suitable for large-scale irrigation or water supply projects. However, recent research efforts have focused on mitigating these limitations by reducing the total cost of PVWPS systems and optimizing their size. This is achieved through the application of suitable metaheuristic algorithms aimed at enhancing overall efficiency and increasing pumping capacity^6,7^.

To enhance PVWPS efficiency, photovoltaic systems are optimized to operate close to their maximum power point (MPP), achieved through the control of the maximum power point tracker (MPPT). Constant Voltage (CV)^8^, Incremental Conductance (IC)^9^, and Perturb & Observe (P&O)^10,11^ represent the predominant conventional control methods employed alongside a dc-dc converter to regulate an MPPT. These methods are known for their simplicity and straightforward implementation in traditional MPPT control systems. However, they are susceptible to oscillations around the maximum power point, leading to difficulties in maintaining a steady trajectory and tracking dynamic changes effectively^12^. Moreover, traditional algorithms are capable of tracking only a single maximum power point (MPP) under consistent irradiation conditions^13,14^. Soft computing (SC) optimizers are employed as a viable substitute for traditional methods, aiming to address their limitations and enhance the efficiency of PVWPS. Furthermore, SC optimizers exhibit robust performance under varying system conditions, leading to notable improvements in system accuracy and responsiveness^15–22^.

Numerous studies have focused on optimizing both the water flow rate (*Q*) and power generation from PV panels (*P*_*PV*_). The control of *P*_*PV*_ and *Q* is achieved through MPPT at different operational stages. Given the influence of external factors like temperature, shading, and radiation, the crucial challenge in designing an efficient PVWPS lies in maximizing generated power by accurately tracking the maximum power point (MPP) of the PV array. The authors investigated a direct coupled photovoltaic water pumping system in^23,24^ under stable conditions. In^25^, the starting features of the motor-pump unit linked with photovoltaic panels were examined independently from MPPT analysis. Elgendy et al.^9^ provides a real-world assessment of utilizing the Incremental Conductance (IC) control method in PVWPS. Conversely, Farhat et al.^26^ illustrates MPPT control of an isolated photoelectric pumping system utilizing a single-input fuzzy logic controller based on the constant voltage (CV) algorithm. Bin-Halabi et al.^27^ introduced an MPPPT algorithm utilizing the adaptive neuro-fuzzy inference system (ANFIS). Ahmed et al.^28^ employed various SC optimizers in a PVWPS system with a DC motor, utilizing an analytical model to forecast the optimal duty cycle required to reach the maximum power point across varying temperature and radiation conditions. Altimania et al.^29^ employed an analytical method to attain the maximum power point (MPP) of the PVWPS system, thereby enhancing both its efficiency and output power. Additionally, ANFIS was utilized to enhance the performance of the pumping system. In^30,31^, the ANFIS technique was utilized to determine the optimal operating voltage, leading to improved performance under both dynamic and steady-state conditions. Belhachat and Larbes^32^ combined the ANFIS controller with the Particle Swarm Optimization (PSO) algorithm to train the MPPT of a PVWPS system, which consisted of two PV modules connected to a soft-switching boost converter.

The implementation of advanced optimization algorithms such as the GTO, HBA, and SAO in real-world PVWPS poses several challenges. These challenges are especially significant due to the unique operational requirements and environmental variability inherent in PVWPS. Main areas of concern include hardware compatibility, cost implications, and the calibration requirements necessary to ensure optimal performance. Below is a detailed discussion of these challenges:

- Implementing optimization algorithms like GTO, HBA, and SAO in PVWPS involves substantial computational demands. These algorithms, although designed for efficiency in solving complex optimization problems, require significant resources in terms of processing power and memory.
- PVWPS systems typically use microcontrollers and processors with limited computational capacity to conserve energy. The computational requirements of algorithms like GTO, HBA, and SAO can strain these systems. Real-time optimization of power output or dynamic adaptation to shading conditions may not be feasible on low-power embedded systems commonly used in PVWPS.
- Accurate data inputs such as solar irradiance, temperature, and voltage are essential for these algorithms to function effectively. Incorporating additional sensors that can provide real-time data for optimization may require hardware upgrades. This creates compatibility challenges between existing PVWPS setups and the new components necessary to run complex algorithms.
- Many optimization algorithms are iterative and may take several iterations to converge to an optimal solution. Real-time adaptation in PVWPS is crucial, especially in fluctuating conditions (e.g., partial shading or rapidly changing weather). Ensuring that the hardware can support real-time data acquisition and processing is essential for the success of these algorithms.
- GTO, HBA, and SAO algorithms require sophisticated hardware, including advanced control units, real-time data acquisition systems, and more powerful processors. Upgrading existing PVWPS with such technology increases initial installation costs, particularly in off-grid, remote areas where cost-effectiveness is often the main goal of deploying solar energy solutions.
- Once the system is operational, there may be additional costs associated with maintaining and updating the software to ensure continued optimization of the PVWPS performance. Additionally, troubleshooting and fixing algorithmic errors could require specialized skills, increasing labor costs.
- Running these computationally demanding algorithms may result in increased energy consumption by the control system itself, slightly reducing the overall efficiency of the PVWPS. In low-power scenarios, this could undermine the gains achieved by the optimization.
- In PV systems, environmental conditions such as shading, temperature changes, and fluctuating irradiance can have a significant impact on performance. For example, shading leads to non-linearities in the P–V curve, creating multiple local maxima. GTO, HBA, and SAO are designed to avoid these local maxima, but maintaining this capability in real-world settings requires constant calibration.
- The effectiveness of these algorithms depends on accurate and real-time data from the PV system. However, in many real-world setups, sensor data can be noisy or imprecise, affecting the calibration of the optimization process. Inaccurate input data can lead to suboptimal algorithm performance.
- Over time, system components may degrade, or environmental conditions may change drastically (e.g., seasonal variations). Therefore, these algorithms would need to be recalibrated periodically to ensure their continued effectiveness. This introduces additional complexity in system maintenance and requires skilled personnel capable of performing these recalibrations.

The application of GTO, HBA, and SAO in real-world PVWPS systems offers promising potential for optimization, particularly in mitigating issues like partial shading and maximizing energy extraction. However, challenges such as hardware compatibility, cost implications, and calibration requirements limit their immediate adoption. Addressing these challenges through hardware advancements, cost reduction strategies, and algorithm refinement will be critical for successful implementation.

This study introduces a multi-objective optimization approach for implementing a soft computing (SC) optimization technique in PVWPS. A buck-boost converter is employed to track the maximum power point (MPP) of the PV system under varying climatic and operational conditions. The proposed SC optimizers utilize solar radiation (*G*) and ambient temperature (*T*) inputs to generate the optimal converter duty cycle (*D*_*op*_). Given the complex and nonlinear relationship between these inputs (*G* and *T*) and the output (*D*_*op*_), the resulting mathematical model is intricate. To address this complexity, the system is trained using advanced SC optimizers such as GTO, HBA, and SAO. Subsequently, the analytical performance characteristics of the PVWPS are obtained and compared after implementing various SC optimizers. To enhance system reliability, minimize computational time, and enable rapid response to sudden operational changes, the obtained analytical results from the proposed online SC optimizers are utilized to offline train an adaptive neuro-fuzzy inference system (ANFIS). This ANFIS system is then implemented in MATLAB Simulink, and the analytical and simulation results are compared.

The subsequent sections of the paper are structured as follows: "Description and mathematical modelling of PVWPS" section outlines the description and computational model of the proposed design, followed by the presentation of the design methodology and the proposed SC optimizers algorithms in "Methodology of SC optimizers" section. "Assessment of SC optimizers" section assesses the performance of the SC optimizers. In "Results and discussion" section, the findings are deliberated upon, including a comparison between the analytical and simulation results derived from MATLAB models. Finally, the study is summarized and concluded in "Conclusion and future work" section.

## Description and mathematical modelling of PVWPS

**Fig. 1:**
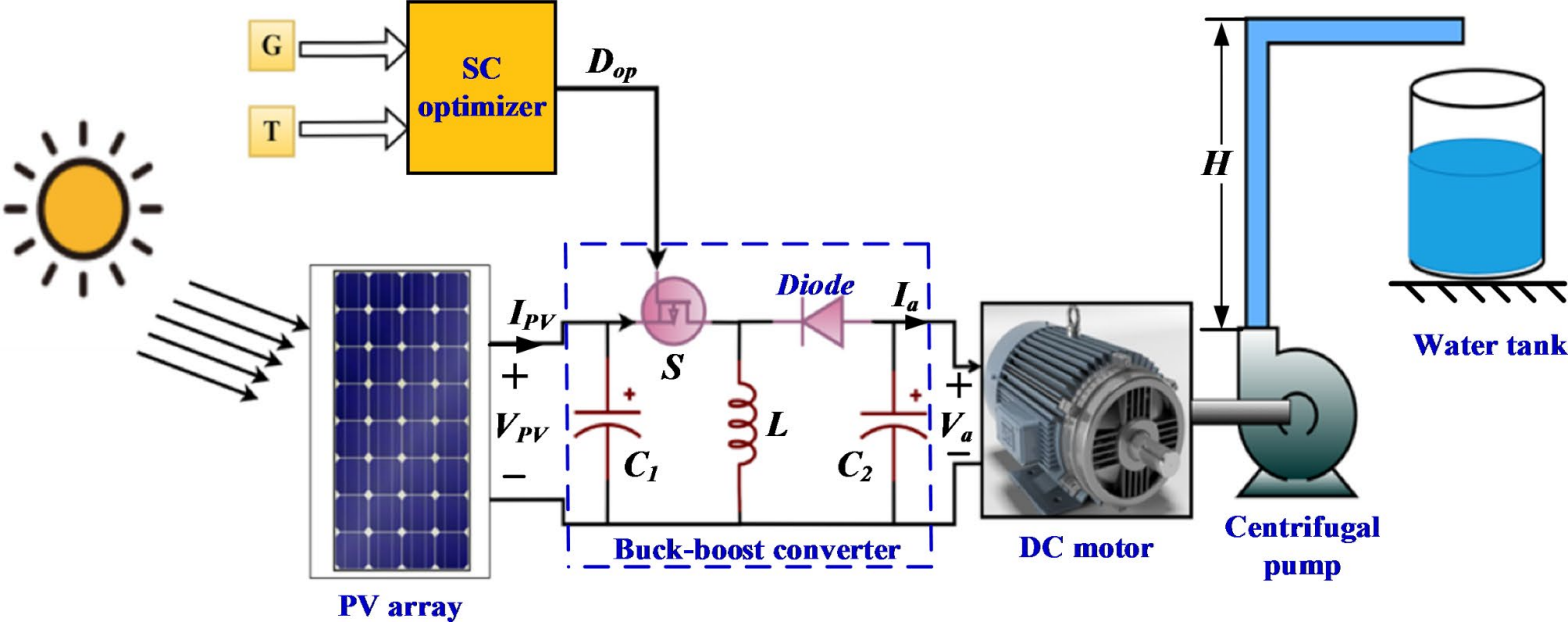
Basic ingredients of the PVWPS.

A photovoltaic water pumping system (PVWPS) usually consists of several basic ingredients; PV panels (PV modules), a DC-DC converter (buck-boost) with SC optimizers, a DC motor coupled with a centrifugal pump, and a storage tank, as depicted in Fig. 1. Steady state computational models have been developed for each element separately and for the whole system as described below.

### PV array modelling

**Fig. 2:**
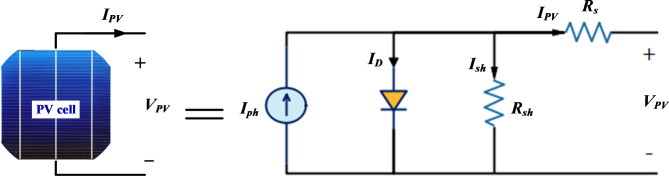
Equivalent circuit of single diode model for a photovoltaic cell.

The photovoltaic generator can be classified as a non-linear power source that is affected by the external environmental conditions such as solar irradiance, ambient temperature, as well as the way the solar cells are connected together. The PV power source can be represented by an equivalent electrical model as illustrated in Fig. 2. This equivalent electrical circuit consists of a single diode (SD) connected in parallel with a current source (*I*_*ph*_), produced by the solar cell as a result of sun light irradiation. Diffusion current (*I*_*D*_) passes through this diode. A resistor (*R*_*sh*_) is connected in parallel to both the diode and the current source (*I*_*ph*_). This resistance represents manufacturing defects as well as the non-perfect nature of the p–n junction. The resistance (*R*_*s*_) is connected in series and is generated as a result of connecting the cells together and the bulk semiconductor materials forming the cell^33^.

From this equivalent circuit both the output current (*I*_*PV*_) and the output voltage (*V*_*PV*_) of the solar cell can be obtained. The current–voltage (*I–V*) characteristics of a single cell can be expressed as in (1)^34–36^.

1 $${I}_{PV}={I}_{ph}-{I}_{o}\left({e}^{\left(\frac{q\left({V}_{PV}+{I}_{PV}{R}_{s}\right)}{ak {T}_{j}}\right)}-1\right)-\frac{{V}_{PV}+{I}_{PV}{R}_{s}}{{R}_{sh}}$$

where *I*_*o*_ is the leakage current of the diode (reverse radiation current), *a* is the diode quality factor (ideality), *q* is the electron charge, *k* is the Boltzmann constant $\left(1.38\times{10}^{-23}\right) J/K$, and *T*_*j*_ is the temperature of the p–n junction in Kelvin (absolute temperature).

**Fig. 3:**
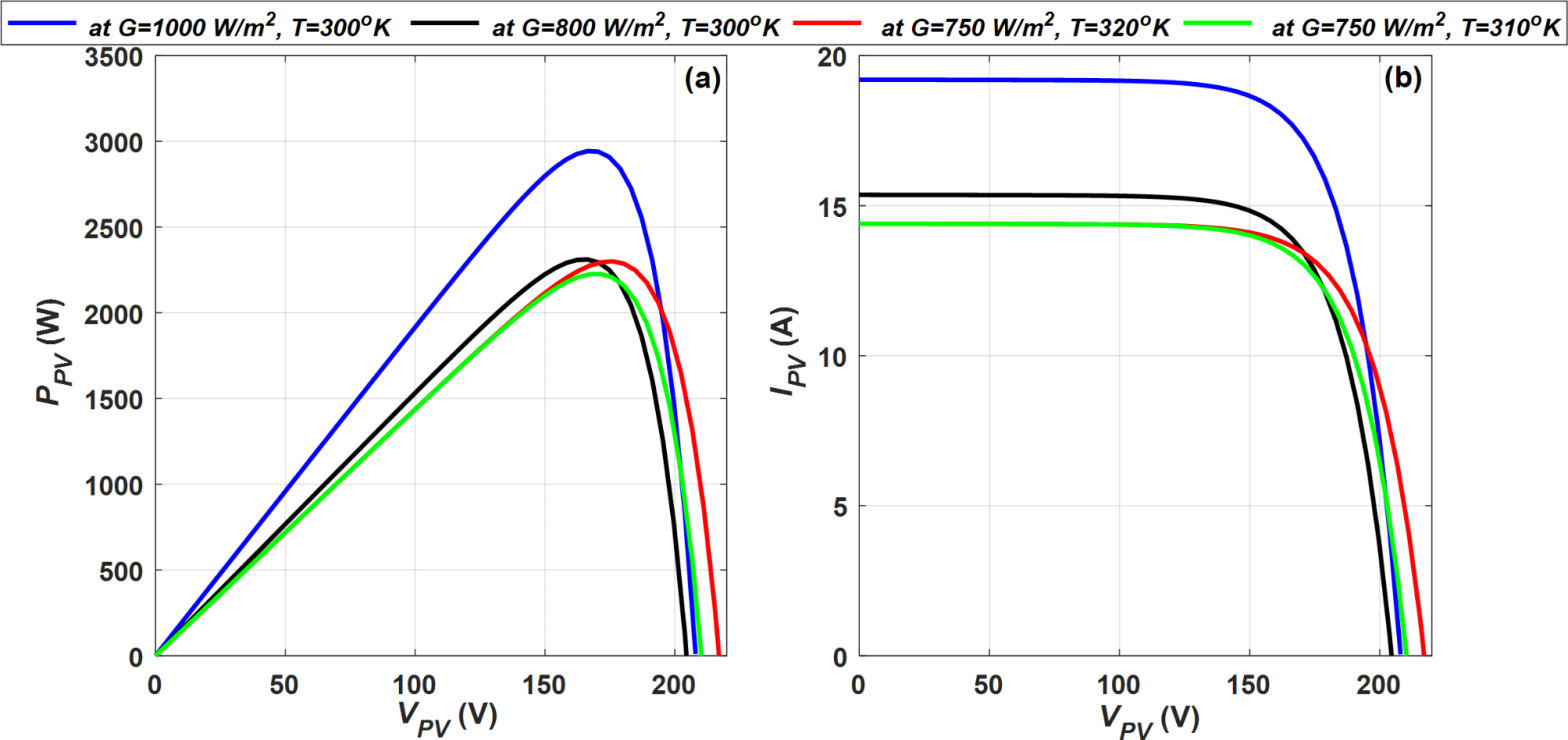
PV characteristic curve (**a**) power (*P*_*PV*_) vs. voltage (*V*_*PV*_), and (**b**) current (*I*_*PV*_) vs. voltage (*V*_*PV*_).

In order to manufacture multiple solar panels, the cells are connected in series, in parallel, or a mixture of series and parallel is made. In this case, the output current (*I*_*PV*_) depends on the number of cells connected in series (*N*_*s*_) and/or the number of cells connected in parallel (*N*_*p*_) expressed as in (2). The simulated characteristics of the PV module at various condition of radiation (*G*) and temperature (*T*) are depicted in Fig. 3. The primary electrical parameters used for the proposed PV module are provided in Appendix A.

2 $${I}_{PV}={N}_{p}\left\{{I}_{ph}-{I}_{o}\left({e}^{\left(\frac{q\left({V}_{PV} + \frac{{N}_{s}}{{N}_{p}} {I}_{PV}{R}_{s}\right)}{akTNs}\right)}-1\right)-\frac{{{N}_{p}V}_{PV}+{N}_{s}{I}_{PV}{R}_{s}}{{N}_{p}{N}_{s}{R}_{sh}}\right\}$$

**Fig. 4:**
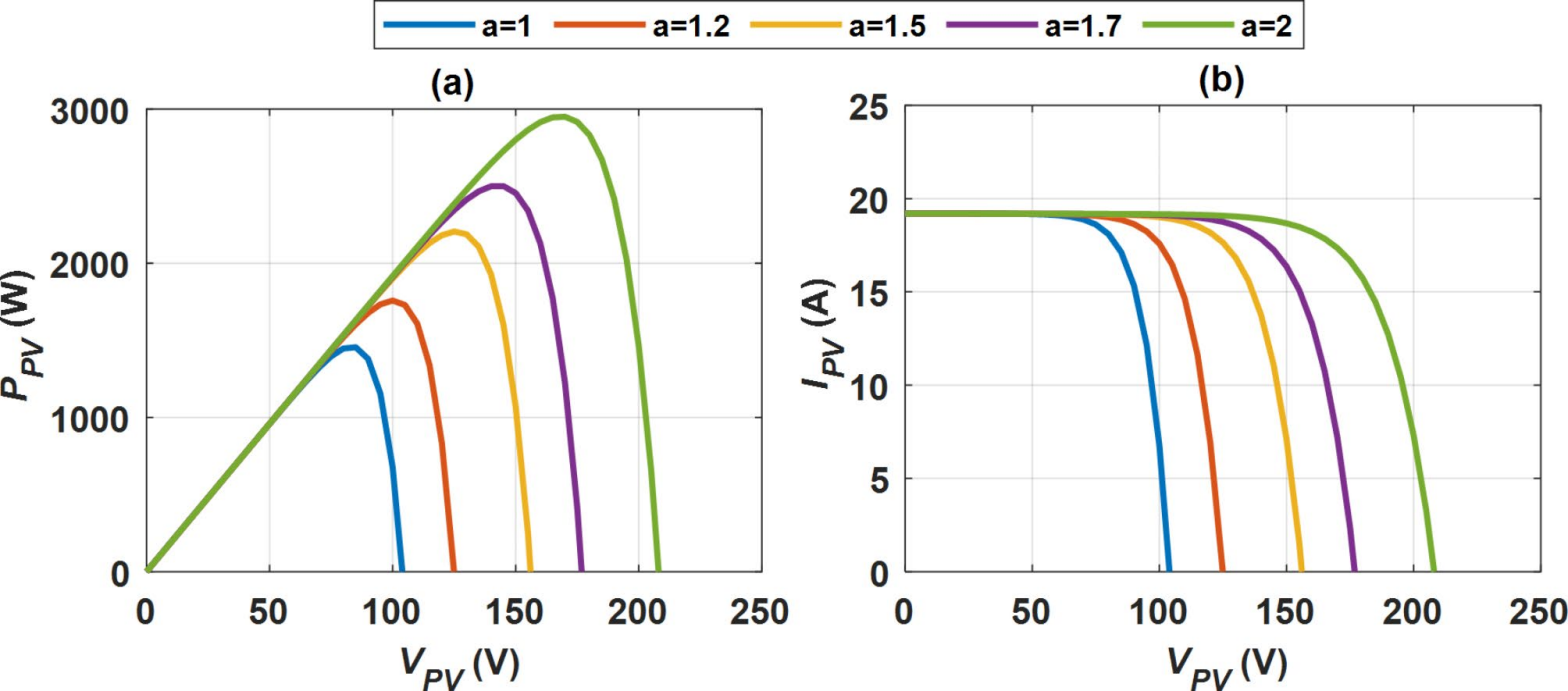
Quality factor impact on PV performance (**a**) power (*P*_*PV*_) vs. voltage (*V*_*PV*_), and (**b**) current (*I*_*PV*_) vs. voltage (*V*_*PV*_).

The quality factor (*a*) in the context of the single diode model for PV cells is a parameter that characterizes the diode’s ideality and efficiency in converting light into electrical energy. It plays a crucial role in modeling the I-V characteristics of PV cells, particularly under varying conditions. The quality factor is influenced by several basic factors, each impacting PV cell performance. Temperature impacts recombination rates, while light intensity affects the interplay between charge generation and recombination. The quality of the material is vital, as defects can significantly impair functionality. Additionally, biasing conditions can change the effective ideality factor, and environmental circumstances such as humidity and dust can cause surface recombination and reduce performance. The impact of variations in the *a* on the performance of a PV system was examined, as illustrated in Fig. 4. As *a* increases, the power curve shifts to the right, and the MPP is higher in terms of both voltage and power output. The peak *P*_*PV*_ of 3000 W occurs at approximately 200 V when the quality factor *a* is set to 2. In contrast, when *a* is 1, the system generates its lowest maximum *P*_*PV*_ of 1000 W at a significantly lower voltage of 100 V. The quality factor signifies enhanced performance of the PV panel, demonstrated by increased MPP and higher voltage levels. At low voltages (near short-circuit conditions), the current begins at approximately 20 A for all values of *a*. However, as the voltage rises, the current drops more significantly for lower values of *a*. For *a* = 2, the current remains higher at elevated voltages, indicating superior performance, whereas for *a* = 1, there is a steep decline in current as the voltage increases. A higher quality factor a enhances the voltage at which the PV panel operates efficiently before the current begins to decrease. The diode quality factor (*a*) greatly affects the performance of a PV system. Increased values of *a* result in enhanced overall performance regarding both power output and operating voltage range. This is crucial in PV system design, as it directly impacts efficiency and energy yield. A higher quality factor diminishes recombination losses in the PV cells, facilitating improved charge carrier collection and optimizing I–V and P–V characteristics. This analysis demonstrates that optimizing a can greatly enhance the performance of PV systems, especially in applications that demand higher voltage and power outputs.

### Buck-boost converter modelling

In this study, a buck–boost converter is used due to its merits including; high efficiency, compact size, low cost and flexible output voltage. In addition to regulating the voltage and the wide range of the input voltage^37^. Considering the ideal scenario by neglecting the losses, the ratio (*w*) between the output current *I*_*m*_ (motor current) and the input current *I*_*PV*_ (PV current) can be calculated as a relationship in the duty cycle (*D*) as represented in (3)^38,39^. The value of *D* ranges from zero to unity.

3 $$\left\{ {\begin{array}{*{20}l} {w = \frac{{I_{m} }}{{I_{{PV}} }} = \frac{{1 - D}}{D}} \\ {D = \frac{1}{{1 + w}}} \\ \end{array} } \right.$$

### DC motor-pump modelling

Nowadays, various types of electric motors are commonly utilized in photovoltaic water pumping systems. DC motors are the most common, especially permanent magnet (separately excited) DC motors. It has several features including simplicity in construction, high reliability, and high efficiency with small loads. In addition, it gives high starting torque, the maintenance requirements are small, and it can be excited directly from the solar panels without resorting to the use of a power inverter. The motor-pump set composes of a centrifugal pump coupled with a DC motor. Under steady state conditions the armature voltage (*V*_*a*_) can be expressed as in (4), in term of armature current (*I*_*a*_), armature resistance (*R*_*a*_), and the counter emf (*E*_*b*_). In addition, the relationship between the electromechanical torque and the armature current is given as in (4)^28^.

4 $$\left\{ {\begin{array}{*{20}l} {E_{b} = k_{t} \times \omega _{m} = V_{a} - I_{a} R_{a} } \\ {T_{e} = k_{t} \times I_{a} } \\ \end{array} } \right.$$

Centrifugal pumps can be used to suit all variable heads, from shallow to high lifts, which can reach 80 m. It also provides higher efficiency when compared to displacement pumps. A centrifugal pump is used in this study, for which power (*P*_*L*_) and torque (*T*_*L*_) can be expressed through the equation mentioned in (5).

5 $$\left\{ {\begin{array}{*{20}l} {P_{L} = 9.81 \times Q \times H} \hfill \\ { T_{L} = K_{P} \times \omega_{m}^{2} } \hfill \\ \end{array} } \right.$$

where *k*_*t*_ is a constant of counter emf (V/rad/s), ${\omega }_{m}$ is the motor speed (rad/s), *Q* is the water flow rate (m^3^/s), *H* is total head (m), and *k*_*P*_* is* a constant of load torque.

When the system is operating under steady state conditions, the motor torque (*T*_*e*_) is equal to the load (pump) torque (*T*_*L*_). So, The PV power and DC motor loss power as a function of duty cycle are expressed in (6). Also, the water flow rate *Q* of the PVWPS can be represented as in (7) depending on the operating point.

6 $$\left\{ {\begin{array}{*{20}l} {P_{PV} = V_{a} w \left[ {\frac{{N_{p} \sigma_{1} }}{{N_{s} R_{sh} }} - I_{o} \left( {e^{{\frac{{ - q\sigma_{1} }}{{a K T_{j} N_{s} }}}} - 1} \right) + GN_{P} I_{ph} } \right] } \hfill \\ {P_{loss} = P_{PV} - K_{P} \omega_{m}^{3} } \hfill \\ {\sigma_{1} = - w V_{a} - \frac{{N_{s} R_{s} }}{{w N_{p} }} } \hfill \\ \end{array} } \right.$$

7 $$Q= {\eta }_{P} {K}_{P}\frac{{\left({V}_{a} - {I}_{a}{R}_{a}\right)}^{3}}{9.81 x H x {k}_{t}^{3}}$$

where *η*_*P*_ is the pump efficiency. The basic parameters of the motor-pump set used in this study are provided in Appendix A.

## Methodology of SC optimizers

**Fig. 5:**
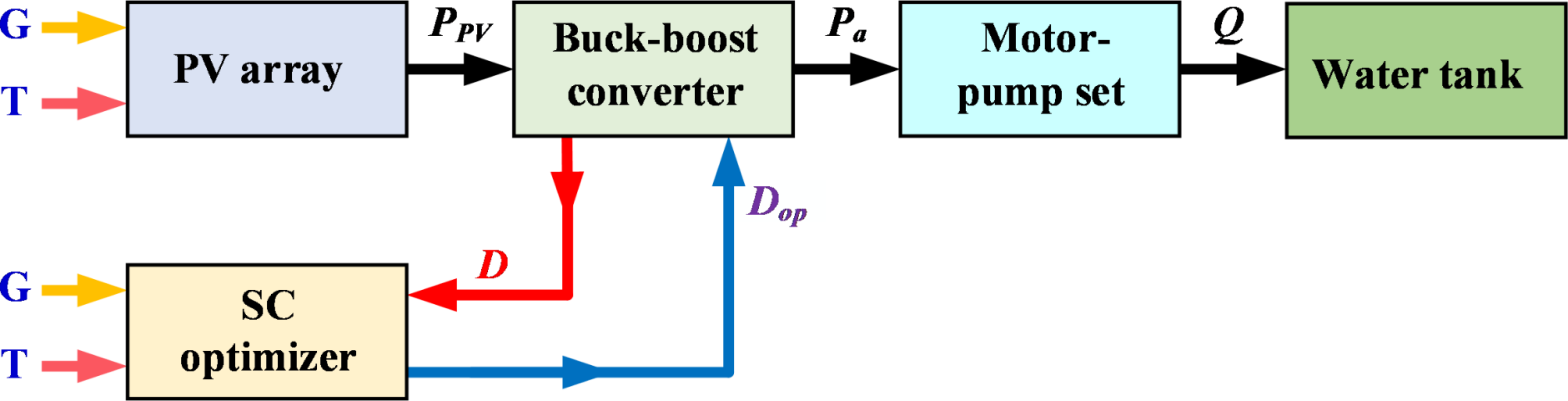
Block diagram of the proposed SC optimizers for PVWPS.

Soft computing (SC) optimizers are used to maximize the output power of the photovoltaic panels. They provide accuracy and speed in obtaining the optimal duty cycle (*D*_*op*_) considering unexpected changes and disturbances occurring in different environmental conditions. By adjusting both current and voltage of the photovoltaic panels, the optimal duty cycle *D*_*op*_ is achieved, taking into account the continuous monitoring of the ambient temperatures and the solar radiation affecting the PV module. Three soft computing optimization algorithms are used to achieve the *D*_*op*_ which are: Honey Badger Algorithm (HBA), Snake Optimization Algorithm (SOA), and Gorilla Troop Optimization Algorithm (GTO). The required objective to be achieved by applying these SC algorithms is to obtain the optimal duty cycle (*D*_*op*_) that achieves a global maximum power point for the photovoltaic module (*P*_*PV*_) and minimize the losses of the DC motor (*P*_*loss*_). Consequently, the motor efficiency (*η*) will be improved and the water flow rate of the motor-pump set (*Q*) is maximized. The required objective functions used in the proposed algorithms can be represented mathematically as in (8). The proposed optimizers are expressed in the block diagram shown in Fig. 5.

8 $$\left\{\begin{array}{l}{Obj}_{1}=\mathit{Max}\left({P}_{pv}\right)\\ {Obj}_{2}=Min\left({P}_{loss}\right)\end{array}\right.$$

### DC motor constraints

To ensure that the DC motor works safely, several constraints related to the maximum power control system must be taken into account. These constraints are divided into two basic types: equality or inequality constraints. The imposed conditions must be fully satisfied in case of equality constraints, while in inequality constraints, the imposed conditions can be partially fulfilled, taking into account the permissible tolerances that do not disturb the safety of the system. The implementation of these constraints is an urgent need to ensure that the DC motor operates within the safety limits and with high reliability, taking into account the undesirable environmental changes. The constraints at which the DC motor must operate can be expressed as in (9) and (10).

9 $$\left\{ { \begin{array}{*{20}l} {V_{a} - V_{rated} \le 0} \hfill \\ {I_{a} - I_{rated} \le 0} \hfill \\ {P_{a} - P_{pv} = 0} \hfill \\ \end{array} } \right.$$

10 $$\left\{ { \begin{array}{*{20}l} {T_{e} - T_{L} - T_{friction} = 0} \\ {\omega_{m} - \omega_{rated} \le 0 } \\ \end{array} } \right.$$

### Gorilla troop algorithm

It is a type of metaheuristic optimization algorithm. This type of algorithm was created by tracking the social behavior of gorilla troops. A simulation of the behavior of groups of gorillas was carried out to find the optimal solution by sharing information and improving their movement and decision-making^40^. The gorilla troop (GTO) algorithm utilizes the behavior of gorillas led by a silverback as inspiration for its reconnoitering stage. Gorilla and silverback represent the solutions nominated by the algorithm, and it was indicated that silverback is the preferred solution between them.

The algorithm uses three mechanisms to traverse the problem space, the first is migration to unknown locations, through which the total problem space is explored, the second is migration to known locations, through which exploration efficiency can be enhanced, while the last is known as relocating to another gorillas, which aids to avert local optimal points. These mechanisms depend on the coefficient "*p*" and are modeled using an equation to facilitate reconnaissance. Equations (11) and (12) were used to simulate the three mechanisms involved in the reconnoitering stage. All details and symbols are defined in^41^.

11 $${\text{G}}X\left( {t + 1} \right) = \left\{ {\begin{array}{*{20}l} {\left( {UB - LB} \right) \times r_{1} + LB,} \hfill & { rand < p} \hfill \\ {\left( {r_{2} - C} \right) \times X_{r} \left( t \right) + L \times H, } \hfill & {rand \ge 0.5} \hfill \\ {X \left( i \right) - L \times (L \times \left( {X \left( t \right) - GX_{r} \left( t \right) + r_{3} \times \left( {X \left( t \right) - GX_{r} \left( t \right)} \right)} \right),} \hfill & {rand < 0.5} \hfill \\ \end{array} } \right.$$

where

12 $$\left\{ {\begin{array}{*{20}l} {C = F\left( {1 - \frac{It}{{MaxIt}}} \right)} \\ {F = \cos \left( {2 \times r_{4} } \right) + 1} \\ {L = C \times l } \\ \end{array} } \right.$$

**Fig. 6:**
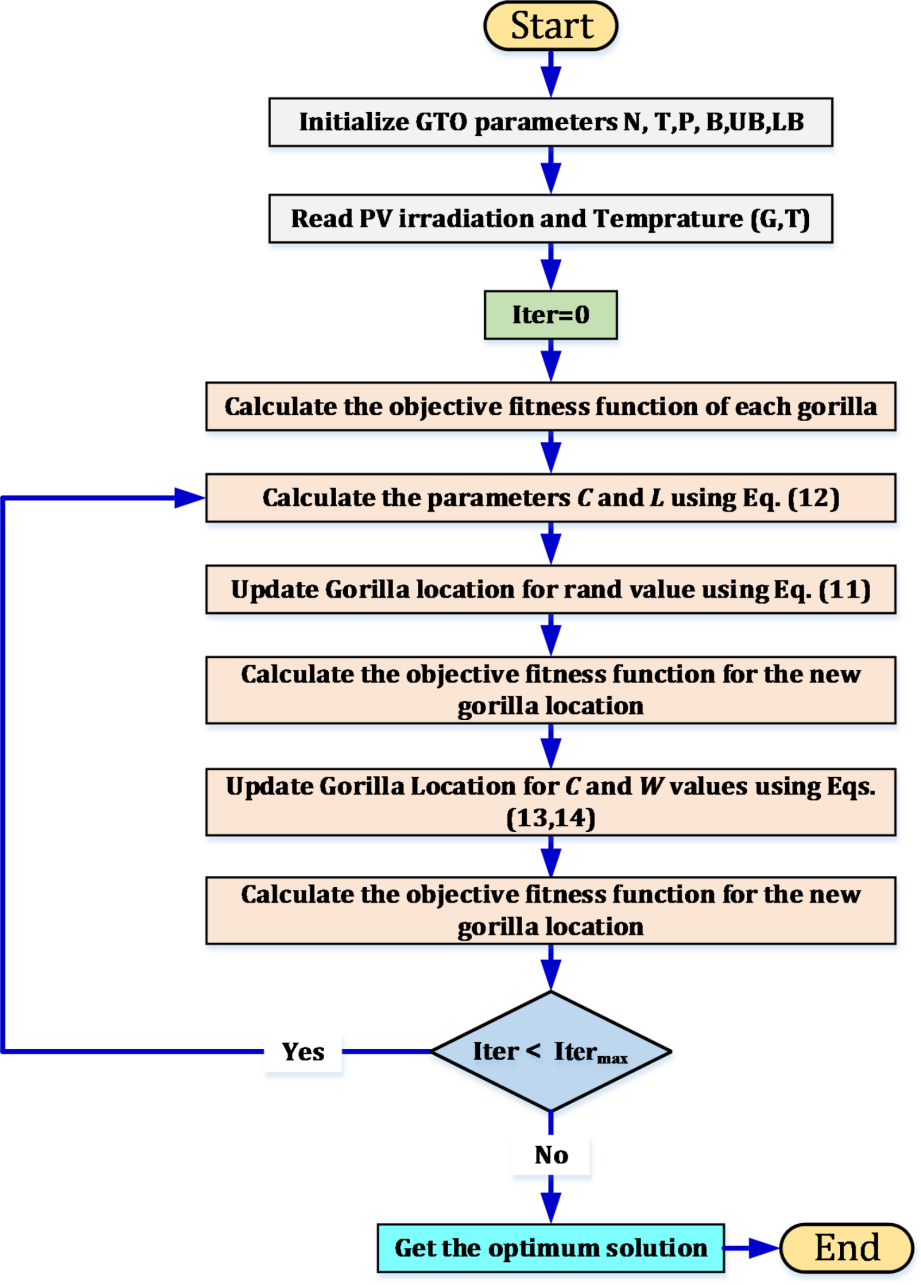
Flowchart for the gorilla troop optimization algorithm.

In the exploitation stage of gorilla troop algorithm, two methods are followed, inspired by the behavior of gorillas, one of which is "Follow the silverback" and the other is "Competition for adult females". In the first method scenario, the silverback is responsible for leading the group and making decisions. When the silverback becomes weak or dies, competition occurs between other males to reach the leadership of the group, in addition to the selection of adult females. The choice between the two methods is based on the value of *C* mentioned in (12). When *C* ≥ *W*, the mechanism "Follow the silverback" is chosen, but when *C* < *W*, the "Competition method for adult females" is the most suitable for selection. *W* is a coefficient placed before the optimization process. A simulation of the follow silverback is made to show its behavior using the Eq. (13), while the behavior of a competition mechanism for adult females is shown through the Eqs. (14) and (15). A summary of the steps describing the gorilla troop (GTO) algorithm is present as illustrated in Fig. 6.

13 $$\left\{ {\begin{array}{*{20}l} {GX \left( {t + 1} \right) = L \times M \times \left( {X \left( t \right) - X_{silverback} } \right) + X \left( t \right) } \hfill \\ {M = \left( {\left| {\frac{1}{N}\sum\limits_{i = 1}^{N} G X_{i} \left( t \right)} \right|^{g} } \right)^{\frac{1}{g}} } \hfill \\ {g = 2^{l } } \hfill \\ \end{array} } \right.$$

14 $$GX \left(i\right)= {X}_{silverback} - \left({X}_{silverback} \times Q-X \left(t\right)\times Q\right)\times A$$

15 $$\left\{ {\begin{array}{*{20}l} {Q = 2 \times r_{5} - 1} \hfill \\ {A = \beta E} \hfill \\ {E = \left\{ {\begin{array}{*{20}c} {N1 , rand \ge 0.5} \\ {N2, rand < 0.5} \\ \end{array} } \right.} \hfill \\ \end{array} } \right.$$

### Honey badger algorithm

The honey badger is one of the mammals known for its courage that takes semi-deserts and African rainforests as a refuge to live in. From the successive follow-up of these mammals while searching for food, a simple optimization mechanism known as the honey badger algorithm (HBA) was discovered. The honey badgers can obtain foraging in one of two ways, either by locating the prey using its strong sense of smell, or by digging to reach the food. In addition, the locations of the beehives can be known by tracking the honey guide birds. These two methods were referred to as the “digging stage” and the “honey stage”, respectively. The honey badgers can move quickly or slowly based on the strength of the scent of the prey. In case of strong scents, its speed is very high and vice versa. The basic control procedures and equations of the honey badger algorithm (HBA) can be summarized as follows^42^:

- *Initialization stage of the HBA*: The number (*N*) and locations of honey badgers are initialized according to Eq. (16).
  16 $${x}_{i} = {lb}_{i} + {r}_{1} \times \left({ub}_{i} - {lb}_{i}\right)$$
  where *r*_*1*_ is a random value ranging from 0 to 1, the lower and upper bounds are expressed in *lb*_*i*_ and *ub*_*i*_, respectively, and *x*_*i*_ is ith location of the honey badger that provides a candidate solution in its population group *N*. honey badger position referring to a candidate solution in a population of N, while and are respectively lower and upper bounds of the search domain.
- *Determine the intensity* (*I*_*i*_): The intensity of the prey scent (*I*_*i*_) depends on the location of the ith honey badger, the farther the honey badger is from its prey, the less intense it is and vice versa. If the intensity of the smell is high, the honey badger will move very quickly, and the lower the intensity, the slower the speed. The relationship between distance and intensity can be represented by applying the inverse square law as in (17).
  17 $$\left\{\begin{array}{c}{I}_{i}={r}_{2}\times \frac{S}{{4\pi d}_{i}^{2}}\\ S={\left({x}_{i}-{x}_{i+1}\right)}^{2}\\ {d}_{i}={x}_{prey}-{x}_{i}\end{array}\right.$$
  where *r*_*2*_ is a random value ranging from 0 to 1, *S* is the concentration intensity which reflects the location of the prey (location of prey), *x*_*prey*_ is the location of the prey (best location), and *d*_*i*_ is the separation between the ith badger and its prey.
- * Update density factor* (*α*): To achieve a smooth transition from the exploration stage to the exploitation stage, the random distribution that changes with time is controlled by the density factor (decreasing factor) (*α*). *α* is updated by using the mathematical Eq. (17) that ensures a gradual decrease with each iteration and therefore the extent of randomness can be reduced over time.
  18 $$\alpha =C\times {e}^{\left(\frac{-t}{{t}_{max}}\right)}$$
  where *t*_*max*_ is the maximum number of iterations, and *C* is a constant larger than 1.
- *Escaping from local optimum*: This procedure and the next two are used to prevent being stuck in local optima areas. This algorithm adds a flag F that modifies the search direction, providing agents more opportunities to properly explore the search field.
- *Updating the positions of agents*: The “digging stage” and the “honey stage” are two detached steps that form the position updating process (*x*_*new*_) for HBA. These stages have various functions, which are described in details below:
  *Digging stage:* The action pattern of the honey badger and the cardioid form are very similar; in addition, the cardioid motion can be simulated by the relationship described in (19). During this stage, the honey badger specifically makes decisions based on this equation.
    19 $${x}_{new}= {x}_{prey} + F \beta {I}_{i} {x}_{prey} + \left(F {r}_{3} \alpha {d}_{i}\times \left|\mathit{cos}\left(2\pi {r}_{4}\right)\times \left[1 -\mathit{cos}\left(2\pi {r}_{5}\right)\right]\right|\right)$$
    where *β* (≥ 1) is the capability of the honey badger to obtain food, and *r*_*3*_, *r*_*4*_, *r*_*5*_,* r*_*6*_, and *r*_*7*_ are random values ranging from 0 to1.

The flag F changes the direction of the search and can be calculated as in (20):

20 $$F = \left\{ {\begin{array}{*{20}l} 1 & { if\; r_{6} \le 0.5} \\ { - 1} & {else} \\ \end{array} } \right.$$

- *Honey stage*: The case in which the honey guide bird guides the honey badger to the beehive can be simulated using Eq. (21).
  21 $${x}_{new} = {x}_{prey}+ F {r}_{7} \alpha { d}_{i}$$

**Fig. 7:**
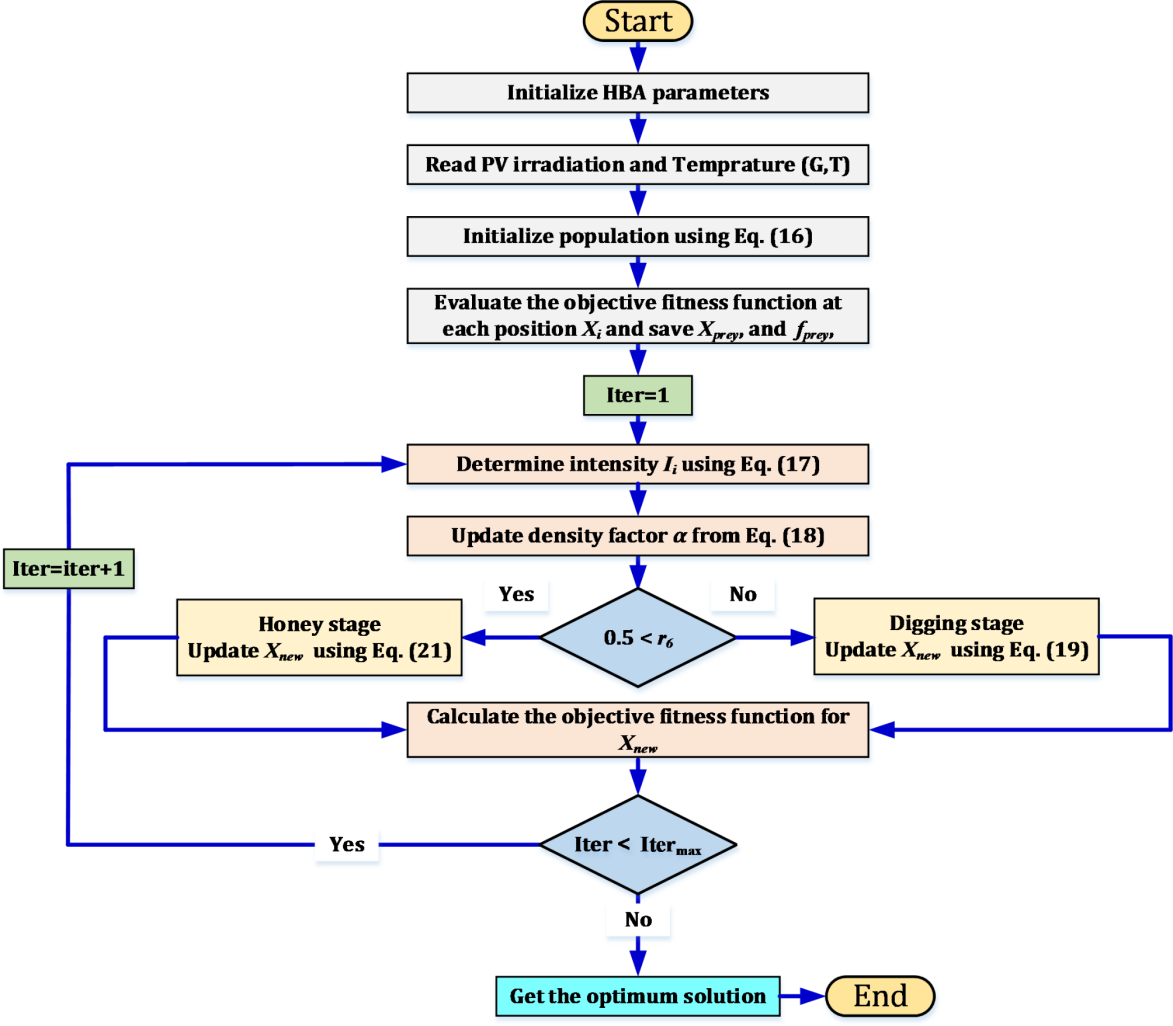
Flowchart for the honey badger optimization algorithm.

The honey badger exploits distance information (*d*_*i*_) to guide its search when it is near its prey location (*x*_*prey*_). Moreover, its behavior is affected by the time-varying searching pattern (*α*), however it can encounter disturbances F while searching. A summary of the steps describing the honey badger algorithm (HBA) is presented as depicted in Fig. 7.

### Snake algorithm

It is an optimization algorithm discovered in 2019. It mimics the activity and movement of a snake in the nature. When applying this algorithm, the most suitable snakes are selected to serve as parents for the next generation, after a re-evaluation of the fitness of each snake separately during movement. After that, the parents are exposed to a number of genetic changes represented in crossover and mutation, which allows the production of a new group of snakes. The basic steps and equations of the snake algorithm is presented as follows^43^:

- *Initialization*: a random population with a consolidated distribution is generated. The Eq. (22) provides the initial population.
  22 $${X}_{i} = {X}_{min} + r \left({X}_{max}- {X}_{min}\right)$$
  where *X*_*i*_ is the location of ith individuals, *r* is a random value ranging from 0 to 1, and *X*_*max*_, *X*_*min*_ are the upper and lower bounds respectively. The population is represented by both males and females, where each type constitutes half (50%) of the population.
- *Defining Temperature and Food Quantity***:** The food quality (*Q*) along with the temperature (*Temp*) can be determined by Eq. (23), if it is possible to determine in advance the best male, the best food location, the best female and the best individual in each group.
  23 $$\left\{\begin{array}{c}Temp={e}^{\left(\frac{-t}{T}\right)}\\ Q={C}_{1}*{e}^{\left(\frac{t-T}{T}\right)}\end{array}\right.$$
  where *t* is the present iteration, *T* is the maximum number of *iterations*, and *C*_*1*_ is a constant.
- *Exploration stage (No Food):* When the value of *Q* is less than the minimum value (0.25), the snakes begin to search for food by choosing any random location and then update their location accordingly. To simulate the exploration phase, equation No. (24) can be followed.
  24 $$\left\{\begin{array}{c}{X}_{i,m}\left(t+1\right)={X}_{rand,m}\left(t\right)\pm {C}_{2} {A}_{m} \left(rand \left({X}_{max}-{X}_{min}\right)+{X}_{min}\right)\\ {X}_{i,f}\left(t+1\right)={X}_{rand,f}\left(t\right)\pm {C}_{2} {A}_{f} \left(rand \left({X}_{max}-{X}_{min}\right)+{X}_{min}\right)\end{array}\right.$$
  where *X*_*i,m*_ and *X*_*i,f*_ is the location of the ith male and female individual respectively, *rand* is a random value ranging from 0 to 1, *A*_*m*_ and* A*_*f*_ is the capability of the male and female to get the food respectively, *X*_*rand,m*_ and* X*_*rand,f*_ is the location of random male and female respectively.
- *Exploitation stage (Food Exists):* If both the temperature (*Temp*) and food quality (*Q*) exceeded the threshold (0.6) (hot) and (0.25), respectively, the snake will not move except in the case of food only. This movement can be expressed in Eq. (25).
  25 $${X}_{i,j}\left(t + 1\right)= {X}_{food} \pm C3 \times Temp \times rand \times \left({X}_{food}- {X}_{i,j}\left(t\right)\right)$$

The location of an individual male or female is represented by *X*_*i, j*_, while *X*_*food*_ indicates the location of the best individuals. The snake mode will be determined according to the threshold value. If the threshold is less than 0.6 (cold), the snake can enter either fighting mode or mating mode.

**Fig. 8:**
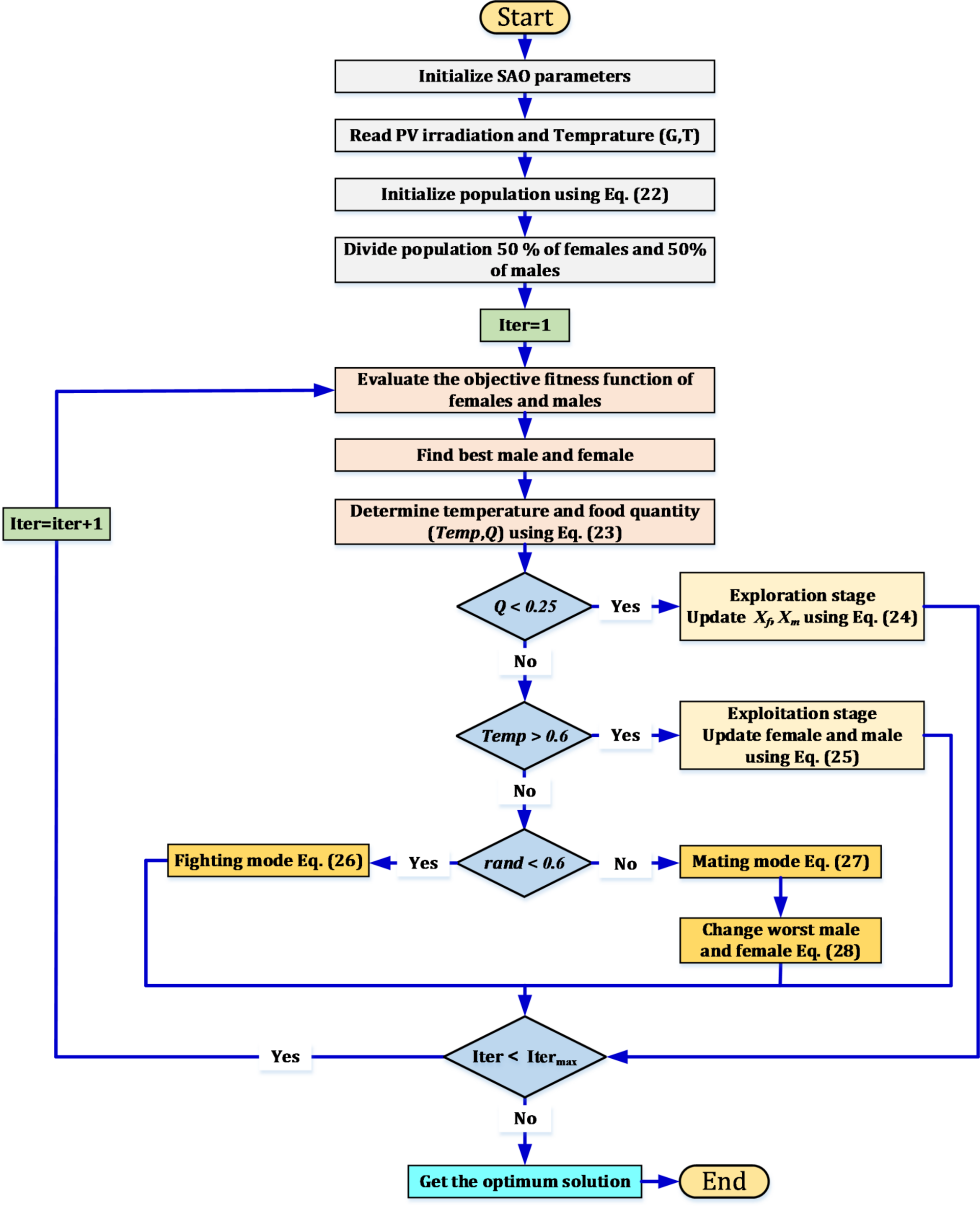
Flowchart for the snake optimization algorithm.

- *Fighting Mode:* Each of males and females updated its location according to its fighting capability as presented in (26).
  26 $$\left\{\begin{array}{l}{X}_{i,m}\left(t + 1\right)={X}_{i,m}\left(t \right)\pm {C}_{3} \times FM \times rand \times \left({X}_{best,f}-{X}_{i,m}\left(t\right)\right)\\ {X}_{i,f} \left(t + 1\right)= {X}_{i,f} \left(t + 1\right)\pm {C}_{3} \times FF \times rand \times \left({X}_{best,m} - {X}_{i,F} \left(t + 1\right)\right)\end{array}\right.$$
  where *X*_*best,f*_ indicates the location best individual of the female group, the male agent’s fighting capability is denoted by FM, while the location of the best male individual is denoted by *X*_*best, m*_, and the position of the ith female individual is represented by *X*_*i,f*_. FF stands for the female agent’s fighting capability.
- *Mating Mode:* Each of males and females updated its location according to its mating capability as presented in (27).
  27 $$\left\{\begin{array}{l}{X}_{i,m}\left(t+1\right)={X}_{i,m}\left(t\right)\pm {C}_{3}\times {M}_{m} \times rand \times (Q \times {X}_{i,f}\left(t\right)-{X}_{i,m}\left(t\right))\\ { X}_{i,f}\left(t+1\right)={X}_{i,f}\left(t\right)\pm {C}_{3}\times {M}_{f} \times rand \times (Q \times {X}_{i,m}\left(t\right)-{X}_{i,f}\left(t\right))\end{array}\right.$$
  where *M*_*m*_ & *M*_*f*_ refers to the mating ability of male and female respectively. In the case of egg hatching, the worst male and female are selected and replaced as presented in (28). A summary of the steps describing the snake algorithm (SAO) is provided as depicted in Fig. 8.

  28 $$\left\{\begin{array}{l}{X}_{worst,m}={X}_{min}+rand\times \left({X}_{max}-{X}_{min}\right)\\ {X}_{worst,f}={X}_{min}+rand\times \left({X}_{max}-{X}_{min}\right)\end{array}\right.$$

### Neuro-fuzzy technique

The adaptive neuro-fuzzy inference system (ANFIS) is a mixed arithmetic model that results from integrating the adaptive learning abilities of neural networks with fuzzy logic which can interpret and represent knowledge. Complicated problems can be solved by neuro-fuzzy systems when neural network and fuzzy logic techniques are combined. ANFIS is characterized by its capability of deal with linguistic and numerical expressions, additionally, the possibility of training them through samples of input–output data. In addition, ANFIS can make accurate decisions, forecasts and improve the performance of any system over time based on its ability to learn from data. It can update and adapt its internal components based on examples of inputs and outputs through the training process. Moreover, ANFIS provides interpretability, robustness against noise and uncertainty in the data and transparency. Ambiguous or uncertain knowledge can be represented by fuzzy logic, causing the system to operate with inaccurate data. Incomplete and noisy information can be handled by generating a fuzzy logic mechanism that enhances the ability of the system to deal with incomplete data in the real world. On the other hand, pattern recognition is done with the possibility of processing a large amount of huge numerical data through neural networks. As a result, complicated real-world problems are effectively modeled and interpreted by adaptive neuro-fuzzy inference systems (ANFIS). In contrast to black-box models (deep neural networks), experts can use fuzzy rules through ANFIS that are easy to understand and modify so that these experts can share their knowledge and better understand the reasoning of the system^29^.

**Fig. 9:**
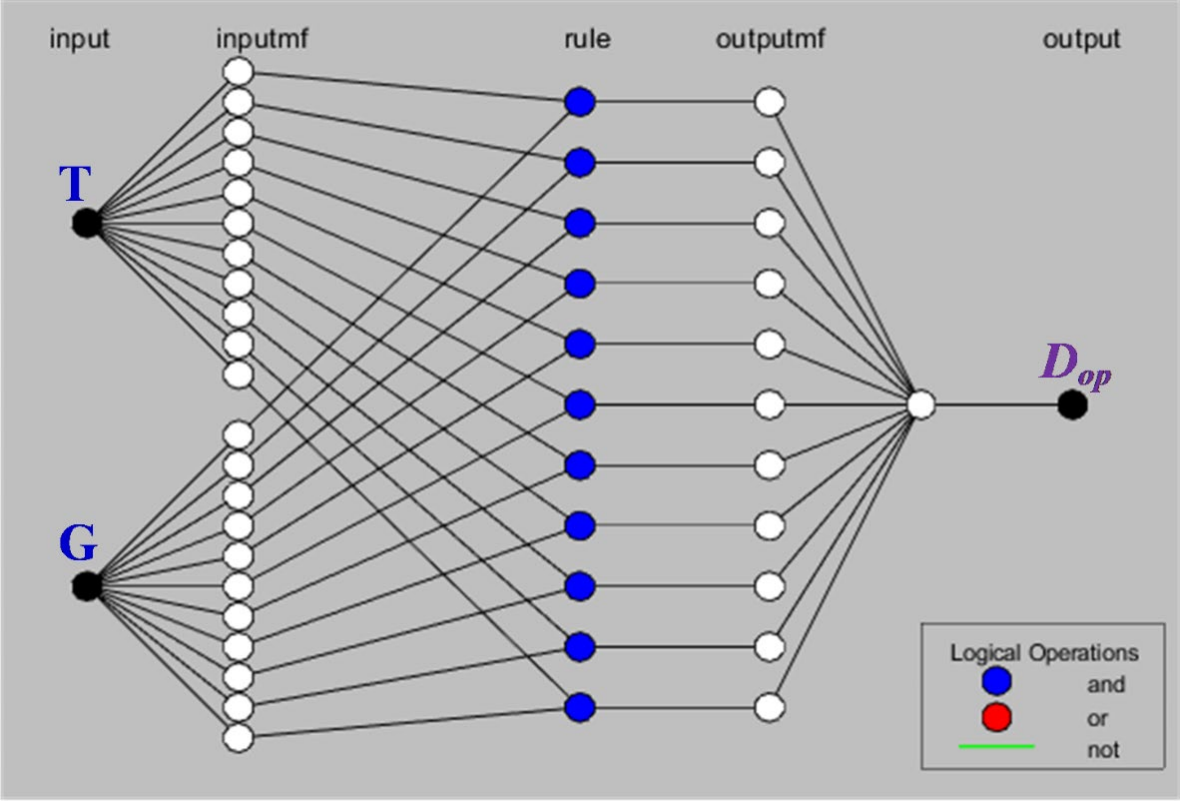
Structure of ANFIS technique.

Membership functions can be considered as an essential element of ANFIS, because they play a vital role in fuzzy inference. It enable the system to identify and manipulate linguistic variables, which leads to easy decision-making and rule-based inference. In addition, it determines the degree of membership and the credibility of the input value for a particular linguistic term. It can also represent mysterious groups and define their shapes and boundaries. The system can explain and interpret uncertain or inaccurate information by assigning membership degrees. There are several forms of membership functions, including Gaussian, trapezoidal, and triangular, in addition they can be built by analyzing data or knowing the domain. Appropriate membership functions are selected based on a combination of factors including data analysis, domain knowledge, and trade-offs between system performance and interpretability. To achieve a specific neuro-fuzzy system, experimentation and iterative refinement must be carried out to find the most appropriate membership functions. In this study a subclustering technique was used to generate membership functions in ANFIS. This technique breaks down the input data into small subsets to generate many local membership functions that pick up more accurate system characteristics. The construction of adaptive neuro-fuzzy inference system is presented as shown in Fig. 9.

**Fig. 10:**
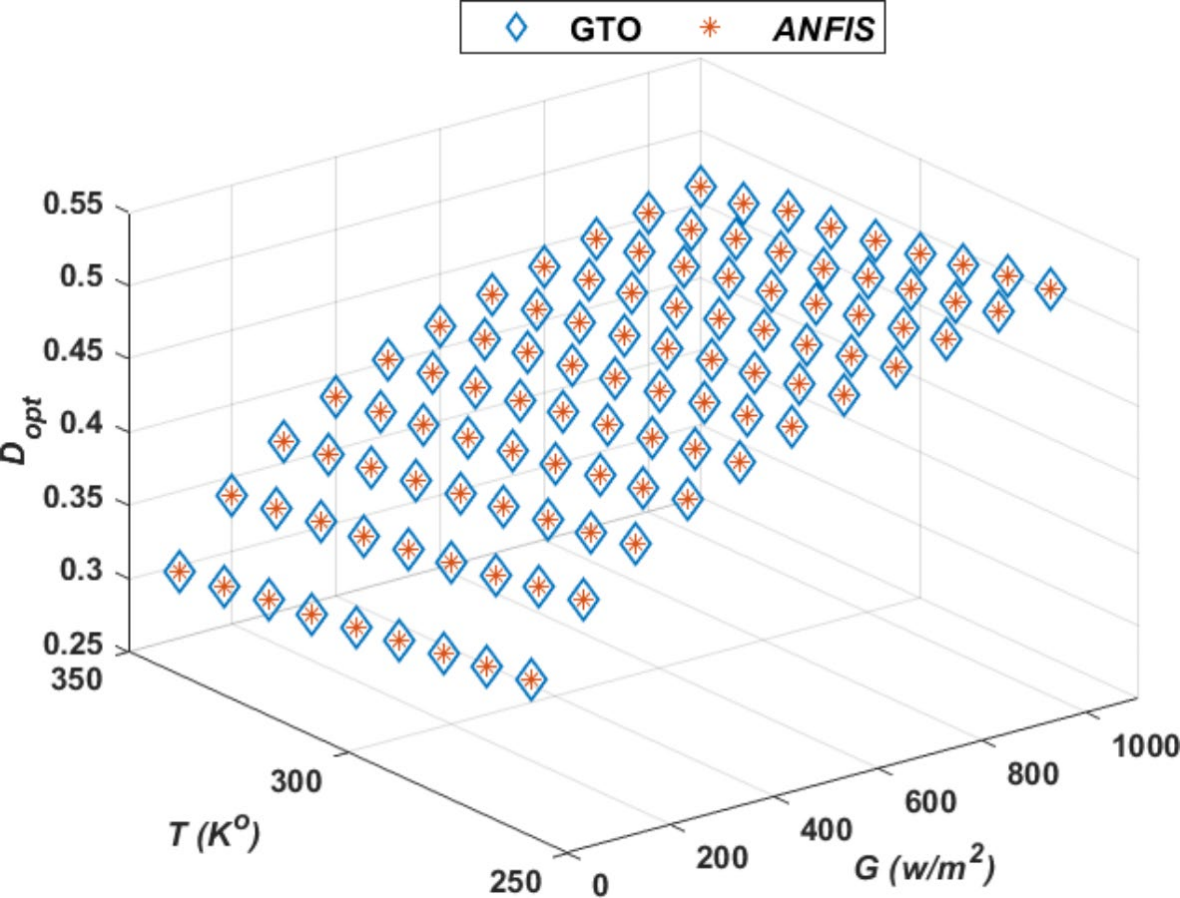
Comparison of *D*_*op*_ data produced by the GTO optimizer and the outputs from ANFIS, taking into account *T* and *G*.

Figure 10 presents the optimal duty cycle (*D*_*op*_) data generated by the GTO optimizer alongside the results from ANFIS, demonstrating the high accuracy achieved during the ANFIS training process. As *T* rises, the *D*_*op*_ shows a slight increase from approximately 0.25 to around 0.55. A similar pattern is observed when *G* increases from 0 to 1000 W/m^2^, with duty cycles steadily rising in both models. Both the GTO and ANFIS models appear to overlap significantly, indicating that both techniques predict very similar values for the *D*_*op*_ over the given range of temperatures and irradiance. The proximity between the *D*_*op*_ of GTO and ANFIS suggests strong agreement between these models and the optimum values. The near-perfect overlap between GTO and ANFIS suggests that these methods provide highly consistent results. The ANFIS model seems to match closely with the theoretical or optimized data (*D*_*op*_), which could indicate a high predictive accuracy of the ANFIS system for this specific application.

## Assessment of SC optimizers

This section evaluates the proposed soft computing (SC) optimization techniques used to achieve maximum power and efficiency in a PVWPS. To assess the effectiveness of the proposed SC optimizers, their performance was evaluated through MATLAB simulations and analytical modeling. The three SC Optimizers were assessed based on their fitness function (*F*_*f*_), computation time (*t*), and 16 benchmark functions to gauge their stability and response speed as indicated in Table 1. The *F*_*f*_ values range from extremely low (1.4 × 10–^79^ for GTO) to significantly higher values (28.94 for SAO). Generally, GTO exhibits very small *F*_*f*_ values, indicating its suitability for high-precision problems, though there is a notable exception with a large negative value of − 12,569.4861, suggesting a possible anomaly. HBA shows a wider range of results, primarily featuring small values but occasionally larger ones (26.94 and 94.975). Similarly, SAO displays a pattern akin to HBA, with several high *F*_*f*_ values (28.94 and 94.975) alongside some smaller ones. This implies that both SAO and HBA may focus on faster convergence, but could be more susceptible to larger errors or extreme outcomes compared to GTO. GTO generally demonstrates faster *t* (around 0.04 to 0.05), indicating its efficiency. However, there is a slight increase in *t* for some extreme results, especially when encountering anomalous *F*_*f*_ values. HBA exhibits somewhat longer *t*, ranging from 0.13 to 0.87, suggesting a potential trade-off between precision and *t* as it manages a wider variety of cases. Similarly, SAO tends to have longer computation times than GTO, particularly when dealing with significantly larger *F*_*f*_ values (0.35 for *F*_*f*_ = 28.94).

**Table 1:** Evaluation of the three SC optimizers.

Benchmark function	GTO		HBA		SAO	
	*F*_*f*_	*t*	*F*_*f*_	*t*	*F*_*f*_	*t*
$\sum {x}_{i}^{2}$	1.4 × 10^–79^	0.043698	4.78 × 10^–28^	0.136131	4.58 × 10^–17^	0.086147
$\sum \left|{x}_{i}\right|+ \prod \left|{x}_{i}\right|$	5.66 × 10^–35^	0.041987	4.046 × 10^–16^	0.162539	9.521 × 10^–07^	0.076589
$\sum_{i=1}^{dim}{\left(\sum_{j=1}^{i}{x}_{j}\right)}^{2}$	3.77 × 10^–62^	0.062688	7.095 × 10^–15^	0.283827	1.711 × 10^–11^	0.146509
$\text{max}(\left|{x}_{i}\right|)$	1.67 × 10^–39^	0.043571	3.969 × 10^–12^	0.133085	1.5013 × 10^–7^	0.080133
$\sum_{i=1}^{dim-1}\left[100{\left({x}_{i+1}-{x}_{i}^{2}\right)}^{2}+{\left({x}_{i}-1\right)}^{2}\right]$	0.48494	0.046239	26.9432	0.152025	28.94077	0.089337
$\sum {\left(\left|{x}_{i}+0.5\right|\right)}^{2}$	0.003059	0.049335	1.86151	0.130562	6.04612	0.076500
$\sum_{i=1}^{dim}i.{x}_{i}^{4}+rand$	0.0002573	0.056216	0.001203	0.235289	0.0006866	0.129470
$\sum - {x}_{i}.\text{sin}(\sqrt{\left|{x}_{i}\right|})$	− 12,569.486	0.049832	− 8278.961	0.163654	-11,936.620	0.098100
$\sum_{i=1}^{dim}\left({x}_{i}^{2}-10\text{cos}(2\pi {x}_{i})\right)+10. dim$	0	0.126148	0	0.869443	94.975	0.232603
$$\begin{aligned} & - 20\exp \left( { - 0.2 x\sqrt {\frac{{\mathop \sum \nolimits_{i = 1}^{dim} x_{i}^{2} }}{dim}} } \right) \\ & \quad - \exp \left( {\frac{{\mathop \sum \nolimits_{i = 1}^{dim} \cos \left( {2\pi x_{i} } \right)}}{dim}} \right) + 20 + {\text{e}} \\ \end{aligned}$$	4.44 × 10^–16^	.069219	7.549 × 10^–15^	0.183654	8.0052 × 10^–7^	0.104131
$\frac{\sum_{i=1}^{dim}{x}_{i}^{2}}{4000}-\prod_{i=1}^{dim}\text{cos}\left(\frac{{x}_{i}}{\sqrt{i}}\right)+1$	0	0.055394	0	0.201008	1.004 × 10^–13^	0.131184
$$\begin{aligned} & \frac{\pi }{dim}\left[ {10\left( {sin\left( {\pi \left( {1 + \frac{{x_{1} + 1}}{4}} \right)} \right)^{2} } \right)} \right. \\ & \quad \left. { + \mathop \sum \limits_{i = 1}^{dim - 1} \left( {\frac{{x_{i} + 1}}{4}} \right)^{2} \left( {1 + 10\left( {sin\left( {\pi \left( {1 + \frac{{x_{i + 1} + 1}}{4}} \right)} \right)^{2} } \right)} \right)} \right] \\ \end{aligned}$$	5.77 × 10^–5^	0.092526	0.06346	0.446544	0.97446	0.239079
$$\begin{aligned} & 0.1\left[ {sin^{2} \left( {3\pi x_{1} } \right) + \mathop \sum \limits_{i = 1 }^{dim - 1} (\left( {x_{i} - 1} \right)^{2} \left( {1 + sin^{2} \left( {3\pi x_{i + 1} } \right)} \right)} \right) \\ & \quad + \left( {x_{dim} - 1} \right)^{2} \left( {\left( {1 + sin^{2} \left( {3\pi x_{dim} } \right)} \right)} \right] \\ &\quad + \sum U_{fun} \left( {x,5,100,4} \right) \\ \end{aligned}$$	3.21 × 10^–5^	0.112491	1.64405	0.462838	2.99249	0.248357
$b{S}_{j }= \sum (({x}^{{\prime} }- a{S}_{j })^6 ) for j=\text{1,2},\dots ..,25$ $o = \left( {\frac{1}{500} + \mathop \sum \limits_{j = 1}^{25} \frac{1}{{j + bS_{j } }}} \right)^{ - 1}$	0.998	0.028223	12.6705	0.657313	0.9980	0.351745
$\sum {(a {K}_{i} - \frac{{x}_{1}\left(b{K}_{i}^{2}+{x}_{2}b{K}_{i}\right)}{b{k}_{i}^{2}+{x}_{3}b{K}_{i}+{x}_{4}})}^{2}$	0.0003074	0.024866	0.0083337	0.125590	0.0005075	0.056051
$4{x}_{1}^{2} - 2.1{x}_{1}^{4}+\frac{{x}_{1}^{6}}{3}+ {x}_{1}{x}_{2}-4{x}_{2}^{2} +4{x}_{2}^{4}$	− 1.031628	0.031861	-0.81578	0.187315	-1.0316285	0.066362

**Fig. 11:**
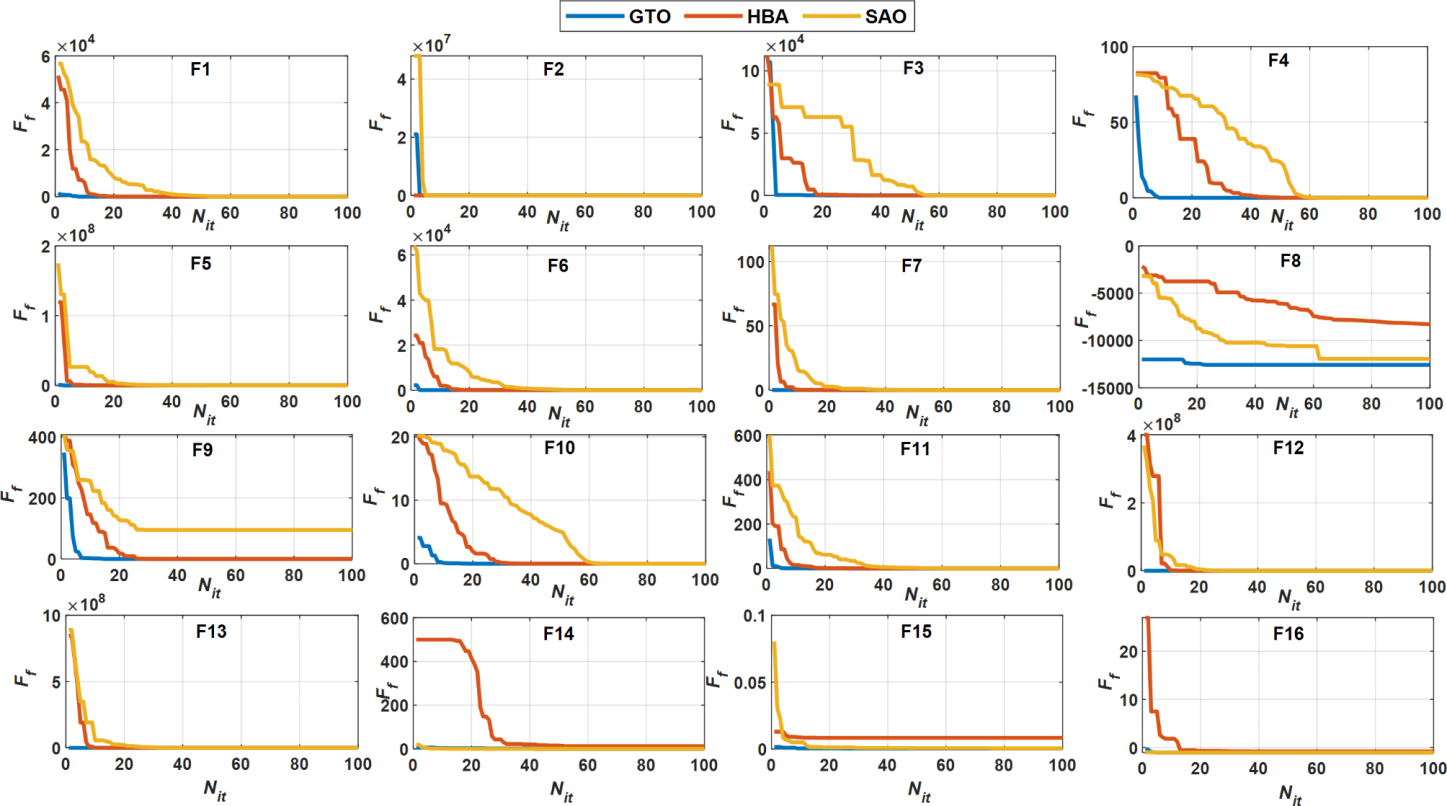
Convergence behavior of three SC optimizers for various benchmark functions (F1 to F16).

Figure 11 demonstrates the convergence behavior of the three methods concerning the number of iterations (*N*_*it*_) and their *F*_*f*_ across various benchmark functions (F1 to F16). In most functions (F1, F2, F4, F5, F7, F9), the GTO method shows rapid convergence within the first 20 iterations, leading to significantly lower *F*_*f*_ values. The HBA method also converges quickly but typically takes a bit longer than GTO, yet often achieves similar results by 40 iterations. The SAO method displays mixed performance across various functions, generally converging slower than both GTO and HBA and sometimes stabilizing at higher *F*_*f*_ values, as seen in F6, F7, and F9. In certain cases (F8, F12, F13), the *F*_*f*_ value decreases but remains relatively high compared to the other methods even after 100 iterations. GTO offers superior performance in terms of both precision (extremely small *F*_*f*_) and speed (shorter times). It consistently surpasses both HBA and SAO regarding convergence speed and accuracy, achieving lower *F*_*f*_ values in fewer iterations for nearly all benchmark functions. These results are consistent with those in the table, further confirming that GTO is the top-performing method among these benchmarks in term of *t* and *F*_*f*_.

**Fig. 12:**
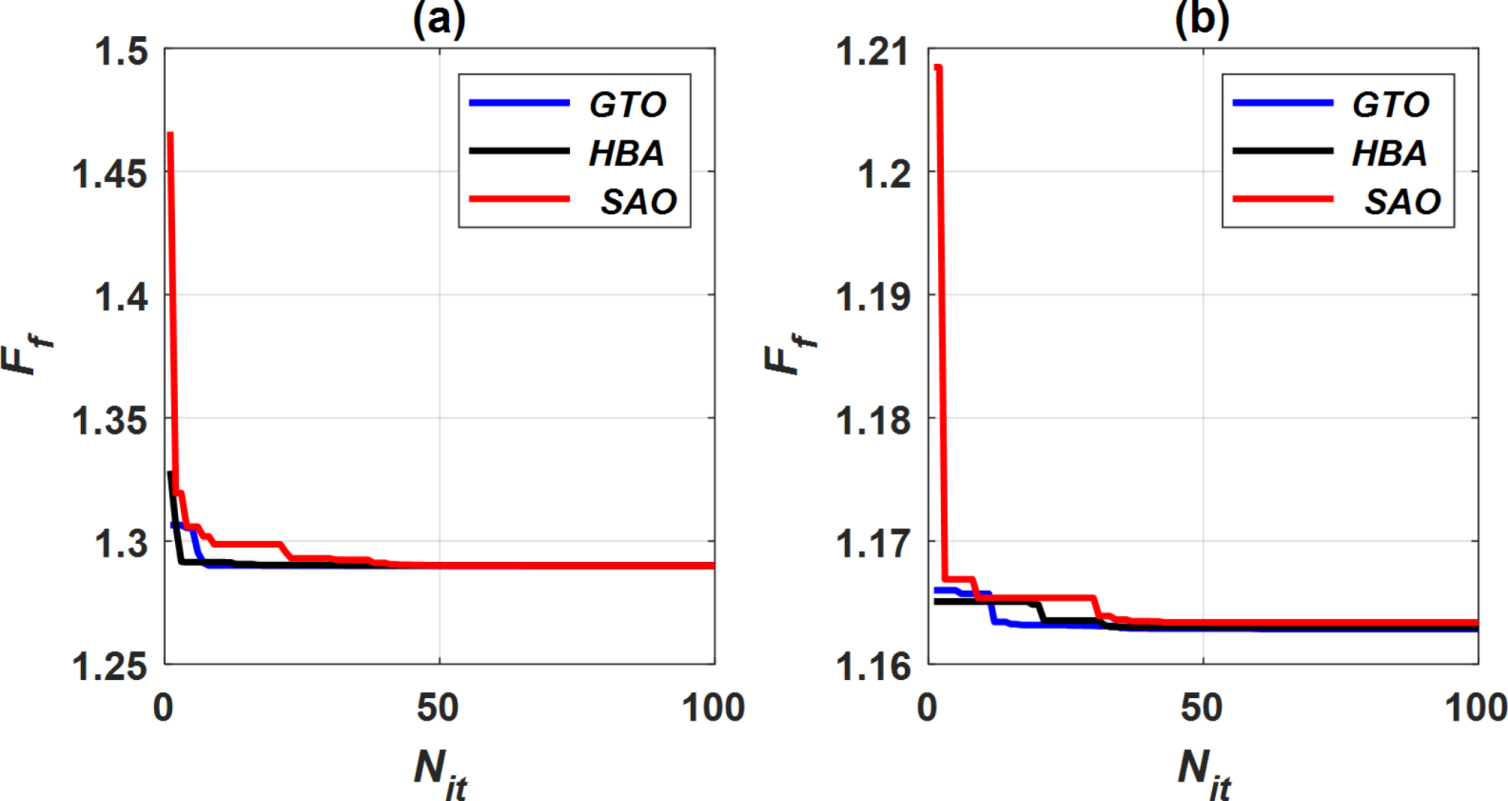
Convergence of different optimization techniques, (**a**)@ *G* = 250 W/m^2^ and *T* = 333 °K, and (**b**)@ *G* = 1000 W/m^2^ and *T* = 333 °K.

The parameters of the proposed GTO, HBA, and SAO techniques are listed in Table 2. To validate the results from the benchmark analysis, the system was tested under different operating conditions by comparing the performance of the three optimization methods: GTO, HBA, and SAO, across various levels of solar radiation (*G*) and ambient temperatures (*T*). The value of *F*_*f*_ was plotted with the *N*_*it*_ for each optimization technique at *G* of 250 W/m^2^ and *T* of 333°K as depicted in Fig. 12a. It was noted that the SAO technique is the slowest way to achieve the desired fitness function value (*F*_*f*_), with the largest number of iterations (48). While the GTO technique achieves the required *F*_*f*_ in the fastest time with the least number of iterations (8). The HBA technique can reach *F*_*f*_ after (17) iterations and an average time. Moreover, the GTO technique achieves the maximum deviation from the required fitness value* F*_*f*_, where it is the lowest deviation at 0.016. In addition, the HBA and SAO techniques have overshoots of 0.03855 and 0.167, respectively.

**Table 2:** Parameters of SC optimizers.

SC technique	GTO	HBA	SAO
Population size	5000	5000	5000
Constants	*p* = 0.03, *β* = 3, and *W* = 0.8	*β* = 6, and *C* = 2	*C*_*1*_ = 0.5, *C*_*2*_ = .05, and *C*_*3*_ = 2
Limits	LB = 0, and UB = 1	*lb*_*i*_ = 0, and *ub*_*i*_ = 1	*X*_*min*_ = 0, and *X*_*max*_ = 1
Threshold	–	–	*Temp* = 0.25, and *Q* = 0.6

The same performance analysis was repeated under different radiation and temperature conditions (*G* of 1000 W/m^2^ and *T* of 333 °K), as shown in Fig. 12b. It was concluded that the GTO technique needs 28 iterations to reach the required fitness value *F*_*f*_, while the HBA and SAO techniques need 34 and 52 iterations to perform same function, respectively. The GTO achieves a deviation from the *F*_*f*_ estimated at 1.16285, which is the lowest when compared with the HBA (1.16302) and SAO (1.16339). Previous comparison cases are summarized in Table 3. Based on the above, it can be concluded that the GTO technique is the most effective proposed optimization method, as it attains the lowest fitness function value (*F*_*f*_) with minimal deviation, in the shortest time, and with the fewest iterations.

**Table 3:** Parameters of various optimization techniques for cases mentioned in Fig. 5.

Parameters	Conditions					
	At *G* = 250 W/m^2^ and *T* = 333 °K			At *G* = 1000 W/m^2^ and *T* = 333 °K		
	GTO	HBA	SAO	GTO	HBA	SAO
Fitness function value	1.29002	1.29003	1.29004	1.16285	1.16302	1.16339
Time (s)	53.774599	57.860599	57.124595	53.995157	57.347573	56.539669
No of iterations	8	17	48	28	34	52

## Results and discussion

In this section, the performance characteristics of the proposed photovoltaic water pumping system were investigated under different conditions of temperature (*T*) and solar radiation (*G*). The results of the analytical model were compared with the results of the simulation model for the three SC optimizers (GTO, HBA, and SAO). Furthermore, an adaptive neuro-fuzzy inference systems (ANFIS) is trained, integrated with the PVWPS, and simulated using MATLAB Simulink software.

### Analytical and simulation outcomes

**Fig. 13:**
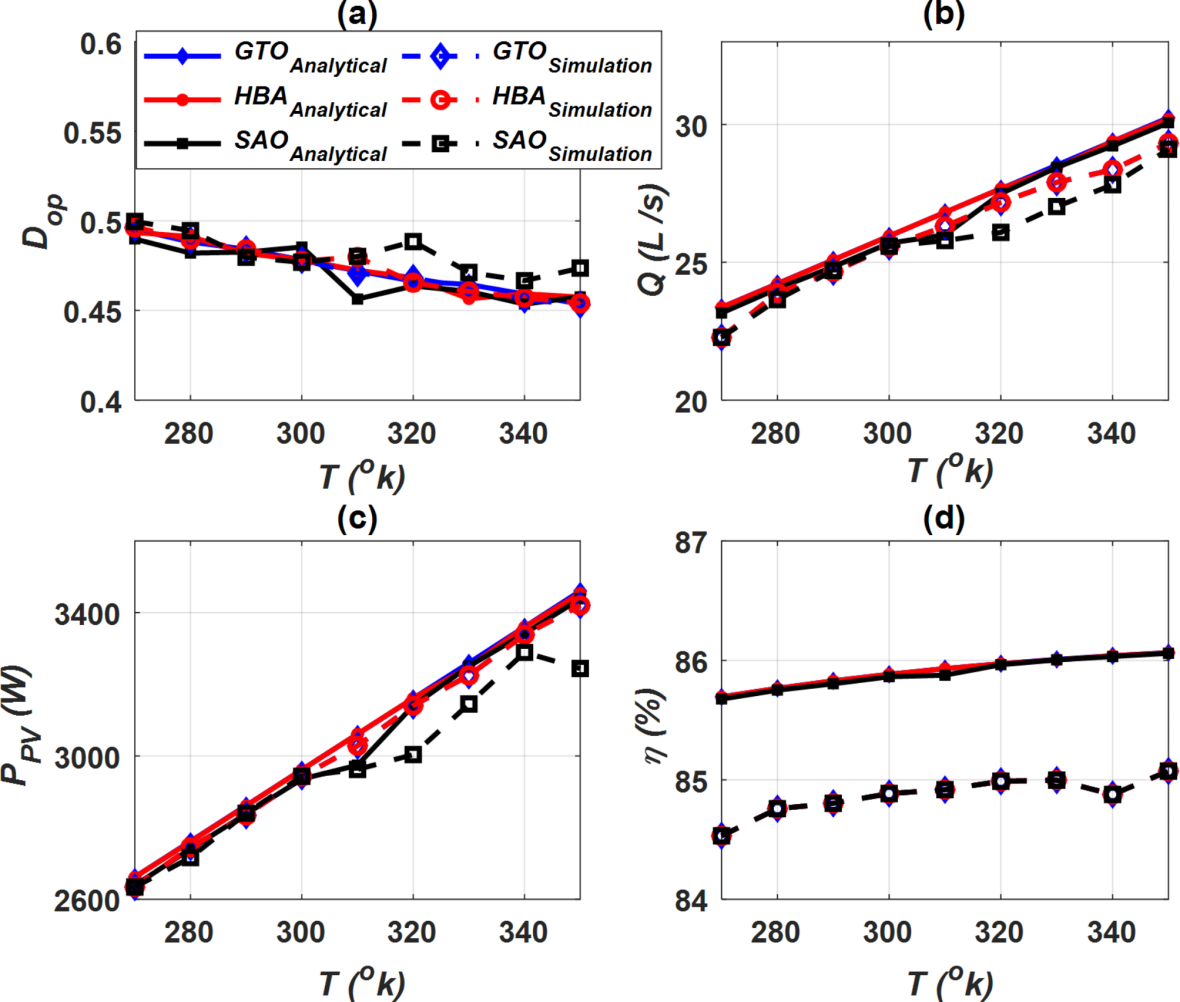
Performance characteristics of PVWPS at constant *G* = 1000 W/m^2^, (**a**) *D*_*op*_ vs. *T*, (**b**) *P*_*PV*_ vs. *T*, (**c**) *Q* vs. *T*, and (**d**) *η* vs. *T*.

**Fig. 14:**
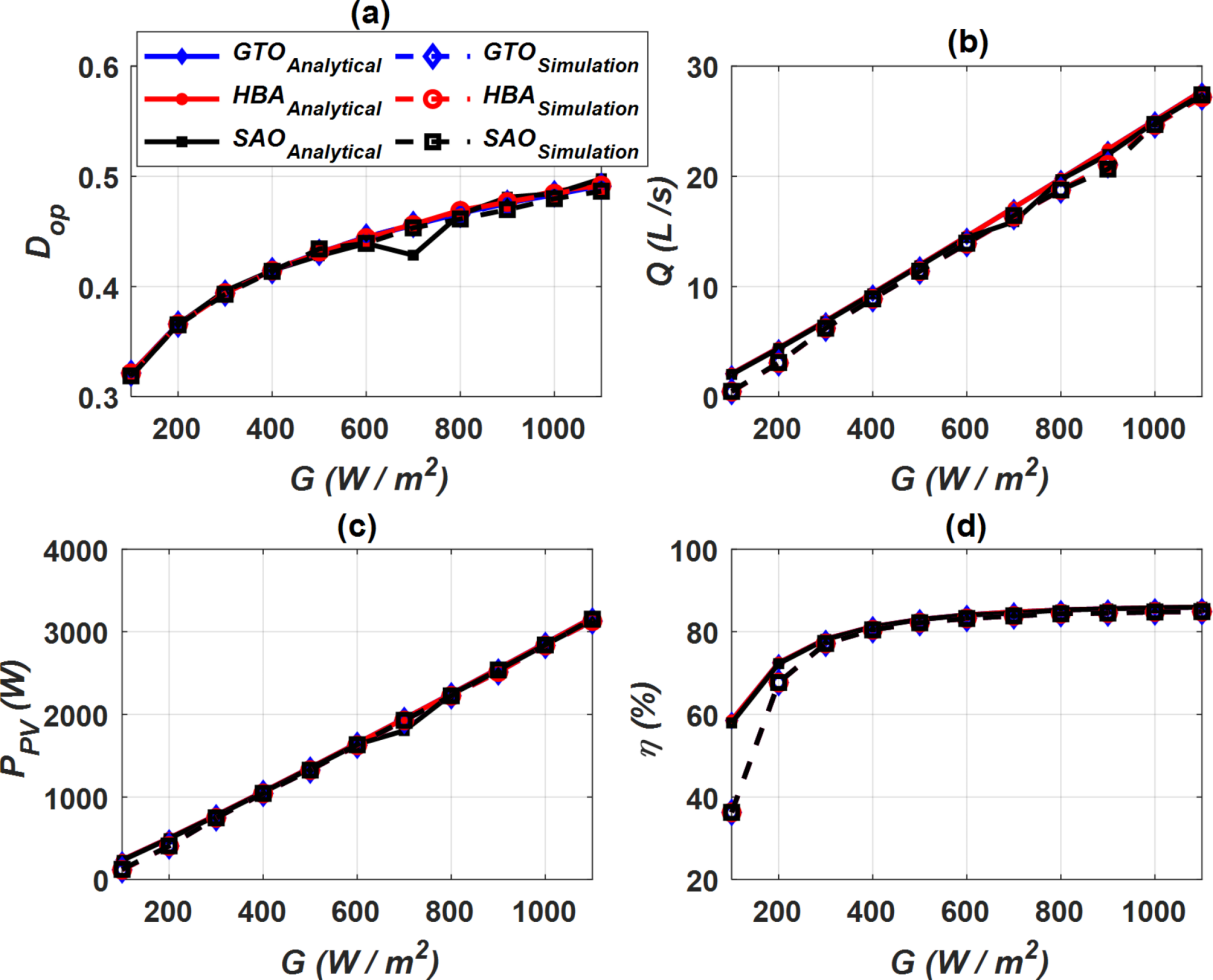
Performance characteristics of PVWPS at constant *T* = 290 °K, (**a**) *D*_*op*_ vs. *G*, (**b**) *P*_*PV*_ vs. *G*, (**c**) *Q* vs. *G*, and (**d**) *η* vs. *G*.

Two cases were considered to study the properties of PVWPS represented in optimal duty cycle (*D*_*op*_), water flow rate (*Q*), output power of PV (*P*_*PV*_) and motor efficiency (*η*). The first case (constant *G*) in which these factors were evaluated at a constant *G* = 1000 W/m^2^ with a change in temperature *T* as shown in Fig. 13, while the second case (constant *T*) was conducted at a constant temperature *T* = 290 °K and a change in radiation *G* as depicted in Fig. 14. Figures 13a and 14a show the variation of *D*_*op*_ of the buck-boost converter for all SC optimizers at constant *G* and *T*, respectively. In the case of constant *G*, the value of *D*_*op*_ decreases as *T* increases, while the opposite occurs when *T* is constant. When *G* changes, the value of *D*_*op*_ increases as *G* increases. *D*_*op*_ achieves its highest value when constant *G* operation is achieved at the lowest value of *T*, while it reaches to its highest value when *T* is constant at the largest value of *G*.

The water flow rate (*Q*) is represented in Figs. 13b and 14b at constant *G* and *T*, respectively. In the constant *G* operation, as the temperature *T* increases, the *Q* increases. Similarly, in the constant *T* operation, as the *G* increases, the *Q* increases. The value of drop in *D*_*op*_, *Q* is very large in the case of constant *T* operation when compared to the constant *G* operation. It is noteworthy that GTO and HBA optimizers provide a higher *D*_*op*_ and *Q* compared to SAO.

The performance of the photovoltaic system was evaluated by making an analysis of the output power (*P*_*PV*_). *P*_*PV*_ is represented in the case of constant *G* and constant *T* in Figs. 13c and 14c, respectively. The *P*_*PV*_ value increases gradually with the increase in temperature *T* when the *G* is constant, and the same thing happens when the *G* is increased in the constant *T* operation. In constant *T* operation, *P*_*PV*_ value is less than its counterpart when the *G* is constant. The findings show that the GTO and HBA optimizers gives a higher *P*_*PV*_ when compared to SAO, which indicates its accuracy in improving the performance of the photovoltaic system. Moreover, the efficiency of the DC motor (*η*) was tested at constant *G* and constant T as depicted in Fig. 13d and 14d respectively. In the constant *G* operation, the efficiency profile is more robust, and its value is higher than its counterpart in the constant *T* operation. The results show that the efficiency of the DC motor *η* is highest when GTO and HBA optimizers are applied.

Overall, the analytical and simulation findings show good correlation. It confirms the effectiveness of the proposed SC optimizers in optimizing the performance of PVPWS. According to analytical and simulation outcomes, GTO and HBA optimizers are the most accurate and efficient optimizers among the proposed SC optimizers. They achieve higher *P*_*PV*_, *Q*, and *η*. It is noteworthy that GTO is distinguished by less computational time and number of iterations compared to HBA.

### SC Optimizers with ANFIS Controller

**Fig. 15:**
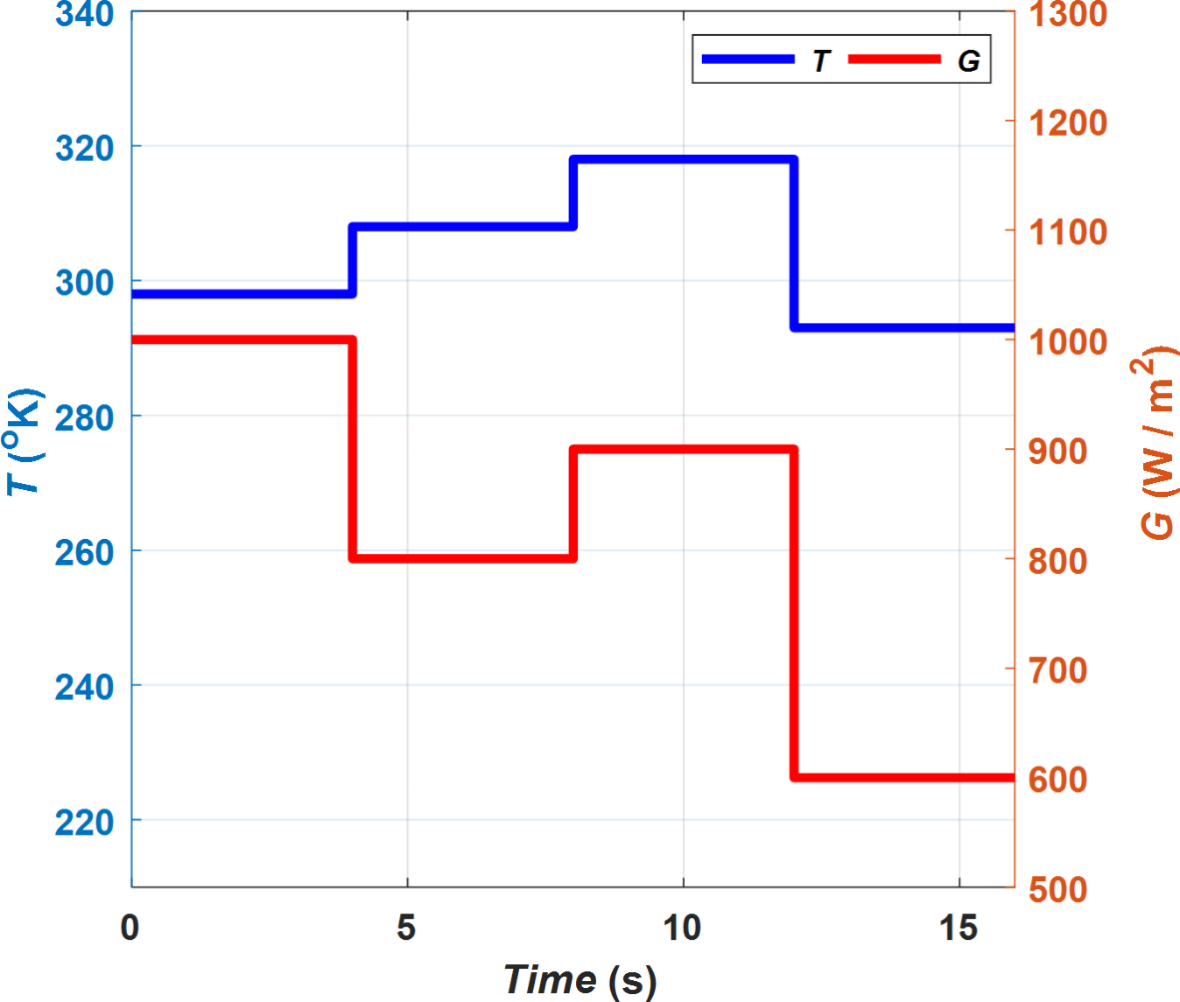
Solar radiation (*G*) and ambient temperature (*T*) of PV generator.

The proposed SC optimizers have been implemented using MATLAB Simulink software, which incorporates ANFIS to enhance the reliability and response time of the PVWPS. In the first stage of this implementation, an adaptive Neuro-fuzzy system is trained offline with inputs of ambient temperature (*T*) and solar radiation (*G*) while its output corresponds to the optimal duty cycle of the buck-boost converter (*D*_*op*_). The proposed SC optimizers are then applied to compare their performance with two conventional P&O and IC control systems^9,11^. The operating conditions of each *G* and *T* for the PV generator is presented with time as depicted in Fig. 15. The simulation outcomes obtained from PVWPS provide a comprehensive evaluation of the effectiveness of the proposed SC optimizers. These outcomes show that the proposed SC optimizers with ANFIS controller significantly improve the performance of the PV generator in terms of accuracy in tracking the MPP, reliability, less computational time, and time response. This results in the operation of the PVWPS with higher efficiency and greater stability and thus can provide a more consistent and uniform water flow rate for the pump system.

**Fig. 16:**
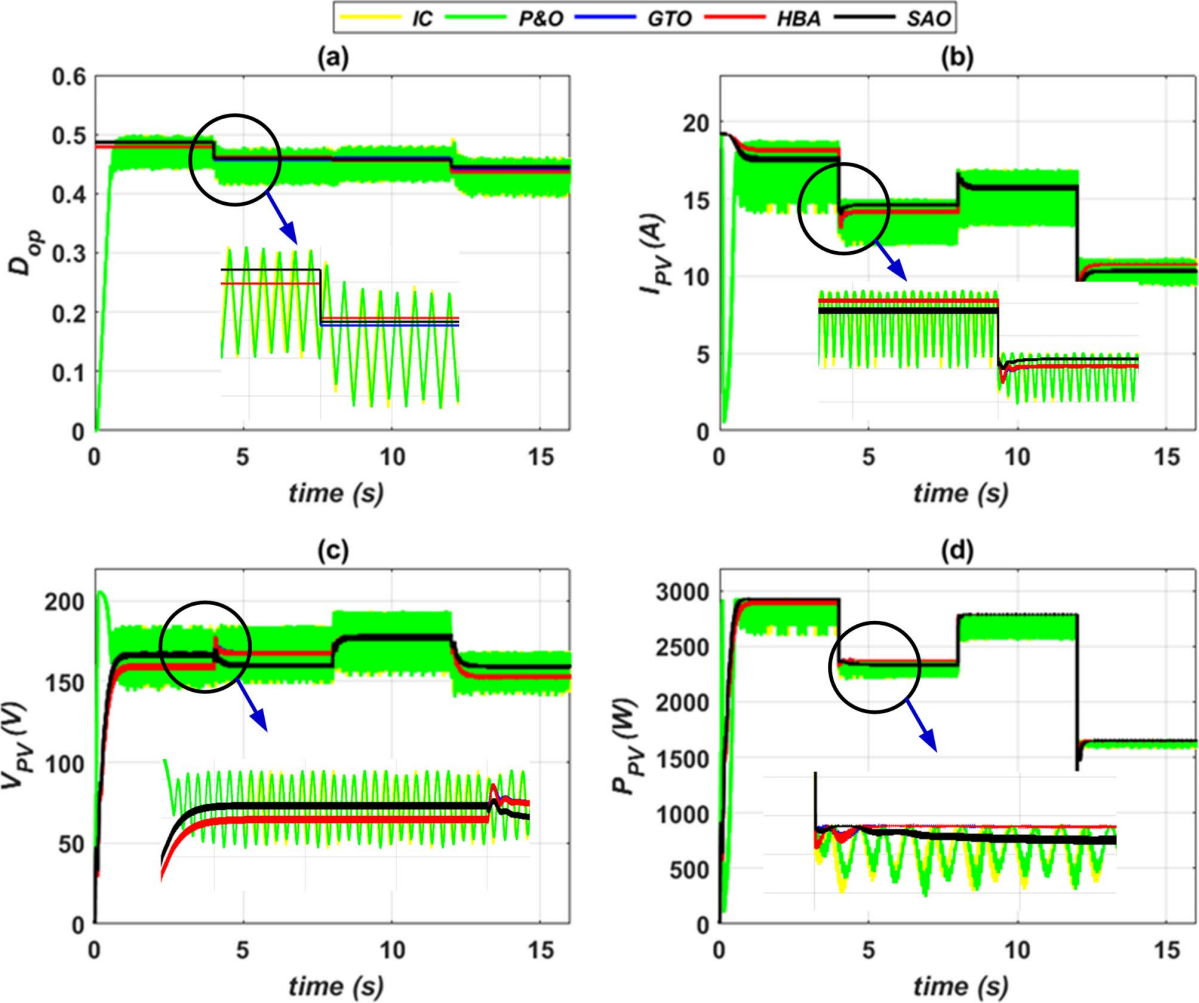
Performance characteristics of PV generator, (**a**) *D*_*op*_ vs. time (**b**) *P*_*PV*_ vs. time (**c**) *V*_*PV*_ vs. time, and (**d**)* I*_*PV*_ vs. time.

The performance of the PV generator with the proposed SC optimizers and conventional MPPT control techniques is demonstrated in Fig. 16. The enhanced performance of the PV generator under the proposed SC optimizers as compared to the P&O and IC algorithms is highlighted. The proposed SC optimizers enable the PV system to accurately track the Maximum Power Point (MPP) with robustness, reduced oscillations, and minimum possible time response.

**Fig. 17:**
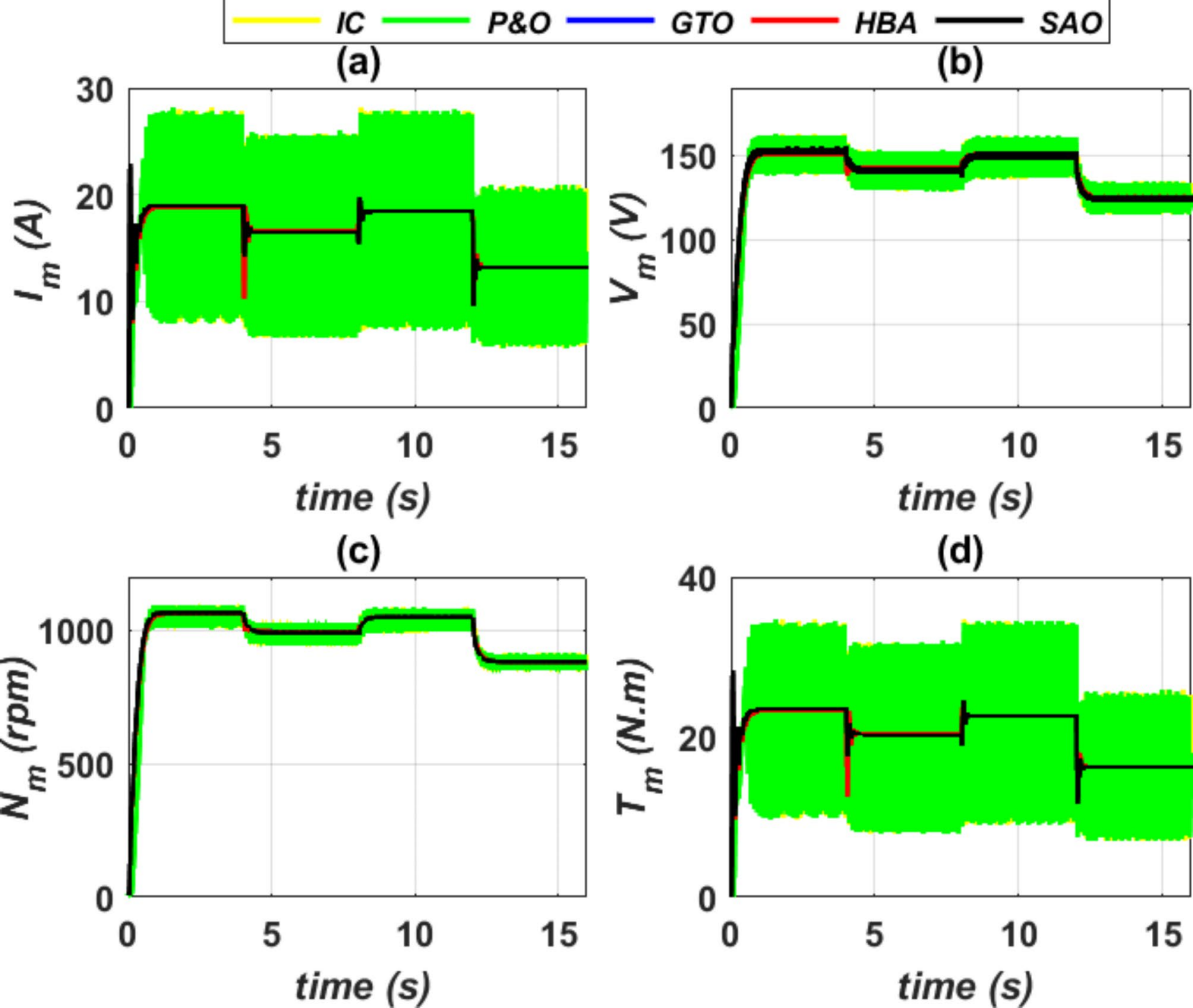
Performance characteristics of DC motor, (**a**) *I*_*m*_ vs. time (**b**) *V*_*m*_ vs. time (**c**) *N*_*m*_ vs. time, and (**d**)* T*_*m*_ vs. time.

The proposed SC optimizers exhibit a smooth dynamic response for the DC motor, as depicted in Fig. 17. The DC motor’s dynamic response in terms of current (*I*_*m*_), voltage (*V*_*m*_), speed (*N*_*m*_) and torque (*T*_*m*_) are illustrated when subjected to variations in the *G* and *T* of the PV generator. It is noteworthy that the DC motor can attain its steady-state value of armature current, electromagnetic torque and speed in the minimum possible time without exhibiting any oscillations. This attribute enhances the stability of the DC motor and guarantees a uniform water flow rate for the pump system.

**Fig. 18:**
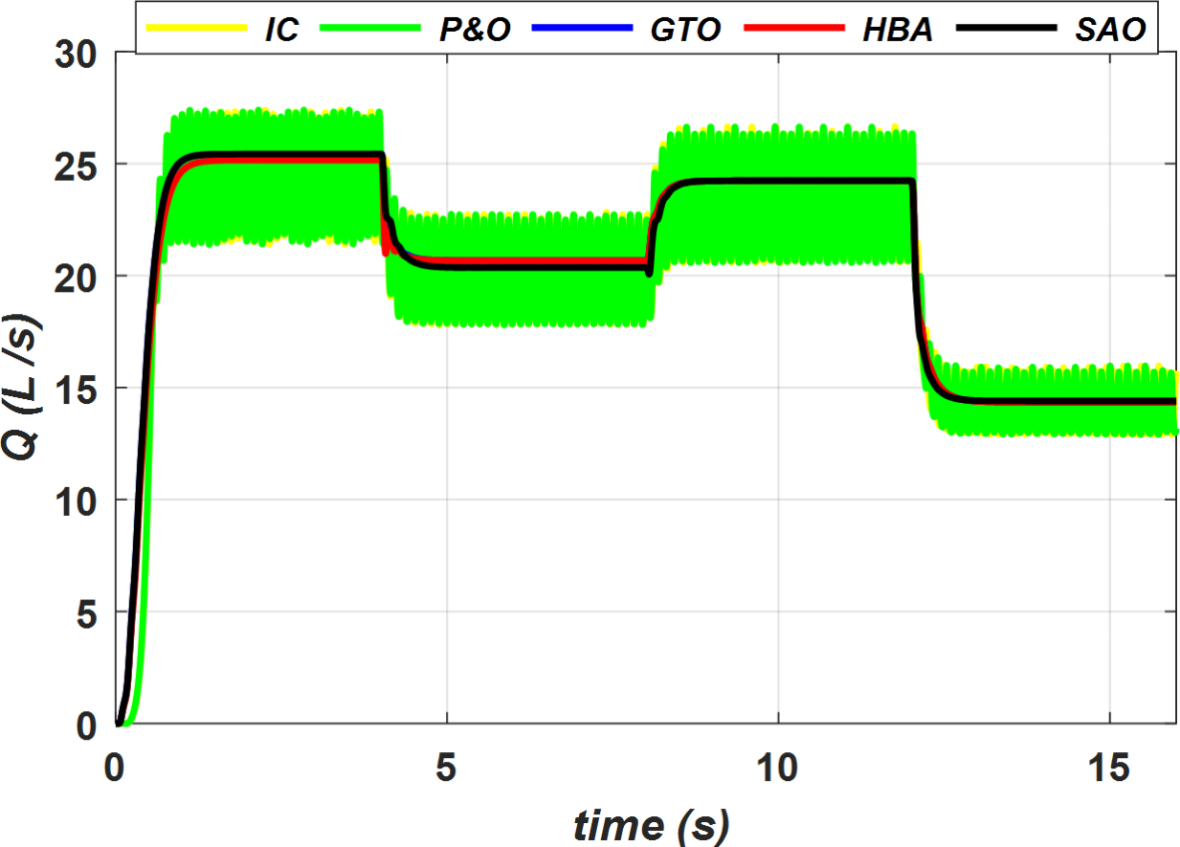
Water flow rate (*Q*) versus time.

The water flow rate of a pump system using the proposed SC optimizer and two conventional MPPT control techniques is illustrated as depicted in Fig. 18. The proposed SC optimizers provide a constant and uniform water flow rate *Q* for specific radiation *G* and ambient temperature *T* conditions, while the conventional MPPT control techniques result in non-uniform water flow rate with high oscillations.

**Fig. 19:**
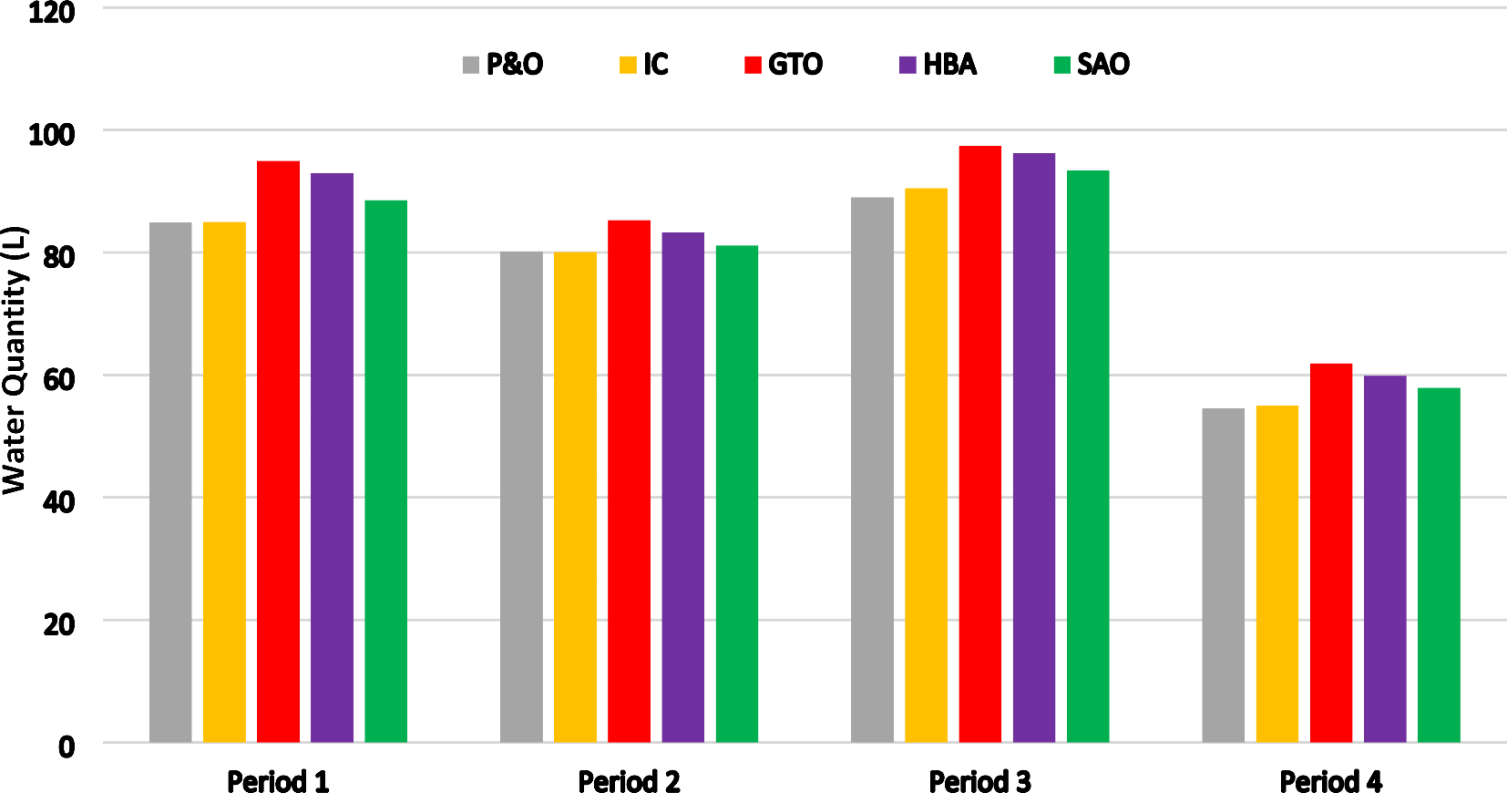
Water quantity (L) versus time.

The total water quantity flow from the water pump over time was 315.14 L and 315.20 L for the MPPT control systems based on P&O and IC, respectively. However, when the proposed SC optimizer was implemented, there was a significant improvement in water flow in the simulation time, with water quantities of 329.50 L, 327.95 L, and 328.28 L for GTO, SAO, and HBA, respectively. These findings confirm that the water pump system’s performance was significantly enhanced, particularly when using GTO as the MPPT control optimizer, compared to the other MPPT control systems. The superiority of the proposed Soft Computing Maximum Power Point Tracking (MPPT) control optimizers over conventional MPPT techniques is demonstrated by Fig. 19, which showcases the pumped water quantities during four different periods under varying conditions of insolation and ambient temperature as period 1 extends from 0 to 4 s, period 2 from 4 to 8 s, period 3 from 8 to 12 s, and period 4 from 12 to 16 s.

The simulation outcomes show that the proposed SC optimizers, integrated with the adaptive neuro fuzzy system (ANFIS), are more reliable and faster in time response than the conventional control systems. Moreover, the proposed SC optimizers exhibit improved stability and accuracy in tracking the MPP under varying conditions of radiation *G* and temperature *T*. The simulation findings demonstrate that the proposed SC optimizers exhibit superior performance in comparison to the conventional P&O and IC control systems. The results of this study demonstrate the effectiveness of the proposed SC optimizers in improving the performance of the PVWPS.

## Conclusion and future work

This paper presents a comprehensive study of various SC optimizers applied to a PVWPS. The analytical and simulation studies evaluate the performance of three proposed SC optimizers and compare them with the conventional P&O and IC control techniques. The results demonstrate that the proposed GTO optimization technique provides the best performance in terms of accuracy, tracking efficiency, system dependency, and convergence time. The simulation study also shows that the proposed SC optimizers, incorporating a Neuro-fuzzy system, significantly improves the performance of the PVWPS, leading to higher output power of the PV (*P*_*PV*_), higher water flow rate (*Q*), higher DC motor efficiency (*η*), and less motor oscillations. Overall, the study highlights the effectiveness of SC optimizers in optimizing the performance of PVWPS under varying environmental conditions.

The analysis and findings of the study suggest several potential research avenues for future investigation, including:

- Carrying out thorough experimental validation to delve deeper into performance of PVWPS in isolated areas.
- Examining latest optimization techniques such as Eel and Grouper Optimizer (EGO), Arctic Puffin Optimization (APO), and Frilled Lizard Optimization (FLO) to determine if they yield significant enhancements in the results achieved in this this PVWPS study.
- Utilizing SC techniques to optimize the integration of energy storage systems (batteries, supercapacitors) into PVWPS.
- Investigating of SC optimization techniques in addressing shading issues in PVWPS.
- Developing distributed optimization techniques, where sub-components of the larger PVWPS are optimized independently or in parallel, can reduce the computational load. Algorithms may need to be adapted to work in a modular framework where each segment of the system is optimized in parallel, rather than optimizing the entire system as a single entity.
- Edge computing could be a promising area of research. By processing data locally at the sensor or inverter level (using embedded optimization algorithms), the need for continuous communication with a central processor could be reduced. This approach could improve scalability by reducing the burden on the central control unit.
- Creating algorithms that can optimize non-uniformly distributed PV arrays, commonly found in building-integrated PV systems (BIPVs), and adjust to varying shading patterns caused by nearby structures or moving objects (such as trees and neighboring buildings) will be crucial.
- An economic analysis is being conducted to compare the proposed SC optimizers with traditional methods.
- In summary, while the Gorilla Troop Algorithm, Honey Badger Algorithm, and Snake Algorithm have shown great potential for optimizing PVWPS, scaling these methods to larger systems or different PV configurations presents significant challenges. Future research must focus on developing scalable optimization techniques that can handle increased complexity, variability, and data processing requirements. Techniques such as hierarchical optimization, hybrid models, edge computing, and machine learning integration offer promising avenues for addressing these challenges. Additionally, adapting these algorithms to grid-connected, hybrid PV-battery, and building-integrated systems opens new opportunities for further innovation in solar energy optimization.

## Electronic supplementary material

Below is the link to the electronic supplementary material.

Supplementary Material 1

## Data Availability

The data used to support the findings of this study are available from the corresponding author upon request.
