# Supplementary material for: Enhanced operation of PVWPS based on advanced soft computing optimization techniques
Source: Sci Rep. 2024 Nov 27;14:29429. doi: 10.1038/s41598-024-80894-1 (PMC11603228; doi:10.1038/s41598-024-80894-1)
Supplement: Supplementary file 1 — Supplementary Material 1 [file 41598_2024_80894_MOESM1_ESM.docx]

**Appendix A**

The basic parameters of the PVWPS used in this study are provided in the Table below.

| **PV module parameters** | | **Motor-pump set data** | |
| --- | --- | --- | --- |
| ***N_s_*** | 300 | ***HP*** | 5 hp |
| ***Np*** | 4 | ***V_a_*** | 240 V |
| ***R_s_*** | 0.00152 Ω | ***I_a_*** | 15.5417 A |
| ***R_sh_*** | 100 Ω | ***N_rated_*** | 1750 rpm |
| ***I_ph_*** | 4.8 A | ***R_a_*** | 0.78 Ω |
| ***I_o_*** | 2.85x10^-5^ A | ***η_P_*** | 0.7 |
| ***a*** | 2 |  |  |
